# Transposable elements modulate human RNA abundance and splicing via specific RNA-protein interactions

**DOI:** 10.1186/s13059-014-0537-5

**Published:** 2014-12-03

**Authors:** David R Kelley, David G Hendrickson, Danielle Tenen, John L Rinn

**Affiliations:** Department of Stem Cell and Regenerative Biology, Harvard University, Cambridge, MA 02138 USA; Broad Institute of Massachusetts Institute of Technology and Harvard, Cambridge, MA 02142 USA; Beth Israel Deaconess Medical Center, Boston, MA 02215 USA

## Abstract

**Background:**

Transposable elements (TEs) have significantly influenced the evolution of transcriptional regulatory networks in the human genome. Post-transcriptional regulation of human genes by TE-derived sequences has been observed in specific contexts, but has yet to be systematically and comprehensively investigated. Here, we study a collection of 75 CLIP-Seq experiments mapping the RNA binding sites for a diverse set of 51 human proteins to explore the role of TEs in post-transcriptional regulation of human mRNAs and lncRNAs via RNA-protein interactions.

**Results:**

We detect widespread interactions between RNA binding proteins (RBPs) and many families of TE-derived sequence in the CLIP-Seq data. Further, alignment coverage peaks on specific positions of the TE consensus sequences, illuminating a diversity of TE-specific RBP binding motifs. Evidence of binding and conservation of these motifs in the nonrepetitive transcriptome suggests that TEs have generally appropriated existing sequence preferences of the RBPs. Depletion assays for numerous RBPs show that TE-derived binding sites affect transcript abundance and splicing similarly to nonrepetitive sites. However, in a few cases the effect of RBP binding depends on the specific TE family bound; for example, the ubiquitously expressed RBP HuR confers transcript stability unless bound to an Alu element.

**Conclusions:**

Our meta-analysis suggests a widespread role for TEs in shaping RNA-protein regulatory networks in the human genome.

**Electronic supplementary material:**

The online version of this article (doi:10.1186/s13059-014-0537-5) contains supplementary material, which is available to authorized users.

## Background

The staggering 45 to 60% of nucleotides in the human genome derived from transposable elements (TEs) remain an enigma in our understanding of the function and evolution of the human genome [1,2]. TEs are sequences capable of propagating by self-replication to new positions in the genome [3,4]. This ability comes in many forms, allowing for classification into a multitude of families [5]. The genomic role of TEs has followed an interesting arc — they were initially described as controlling elements in maize, due to the impact of insertions on local gene expression [6]. As their significance was recognized, it was noted that TEs’ ability to self-replicate meant that a beneficial functional role was unnecessary to explain their conquest of the genome [3,4]. This led to their well-known categorization as junk DNA.

Recent research has revisited the topic of TE impact on gene expression, noting that the dissemination of highly similar sequence accomplished by TEs is a powerful way to link many diverse genomic regions into a regulatory network [7]. In a number of cases, extant TE sequences have integrated with established genomic functions and been co-opted by the genome for critical roles [7,8]. In the most studied paradigm, some TEs contain DNA binding site motifs for transcription factors and have rewired the transcriptional regulatory networks in which these transcription factors function by introducing many new binding sites via their insertions throughout the genome [9-14].

In the substantial portion of the genome transcribed into RNA [15], TE-derived sequences also appear in RNA transcripts where they can interact with RNA binding proteins (RBPs), which also often have preferred binding site motifs [16]. In perhaps the most understood and interesting example, the antisense strand of Alu elements contains motifs that recruit the cell’s splicing machinery and have thus introduced hundreds of novel exons into various protein coding genes [17-19]. Sequence derived from TEs has also been implicated in both degradation [20] and increasing the translation [21] of RNA transcripts. However, the extent to which these examples generalize is unknown, as a comprehensive search for interactions between TEs and RBPs has not yet been performed. Such a search is further justified by the recent appreciation that long noncoding RNAs (lncRNAs), a class of more than 10,000 genes with a rapidly growing list of critical functional roles [22,23], contain TEs at a rate near the high genomic average but in biased proportions of the various individual families, suggesting a possible functional role [24,25].

Crosslinked immunoprecipitation (CLIP)-Seq is the state of the art technique for mapping the direct binding sites of an RBP. It involves crosslinking cells to lock RNA-protein interactions, immunoprecipitating the complexes using an antibody specifically targeted to the RBP, sequencing cDNA reverse transcribed from the captured RNA, and statistically analyzing the aligned sequencing reads [26]. CLIP-Seq has been applied to dozens of RBPs to study splicing regulation [27-29], translation efficiency [30-32], and explore RBPs mutated in neurological disorders [33]. These studies largely focused on uniquely mapping reads and ignored repetitive sequences, leaving the extent of RBP binding to TEs unexplored.

Here, we surveyed evidence for RBP binding to TE-derived RNA sequence in a collection of 75 CLIP-Seq experiments on 51 RBPs performed in human cells. We processed all datasets using a standardized CLIP-Seq analysis pipeline. In these data, RBP interactions with TE-derived sequences were widespread, and we detected hundreds of specific pairwise interactions. Alignment coverage clustered on specific regions of the TE consensus sequences. From these high coverage regions, we extracted a diversity of TE-specific motifs that extensively characterize the *in vivo* binding preferences of the RBPs. The presence of CLIP-Seq coverage and conservation at nonrepetitive instances of these motifs suggest that the TEs appropriated existing binding preferences of the RBPs. RBP binding to TE-derived sites influenced RNA abundance and splicing to a comparable extent as binding to nonrepetitive sites in RBP knockdown experiments. Altogether, our comprehensive meta-analysis suggests a widespread role for TEs in shaping post-transcriptional RNA-protein regulatory networks in the human genome.

## Results

### CLIP-Seq alignments are enriched in specific transposable elements

**Figure 1:**
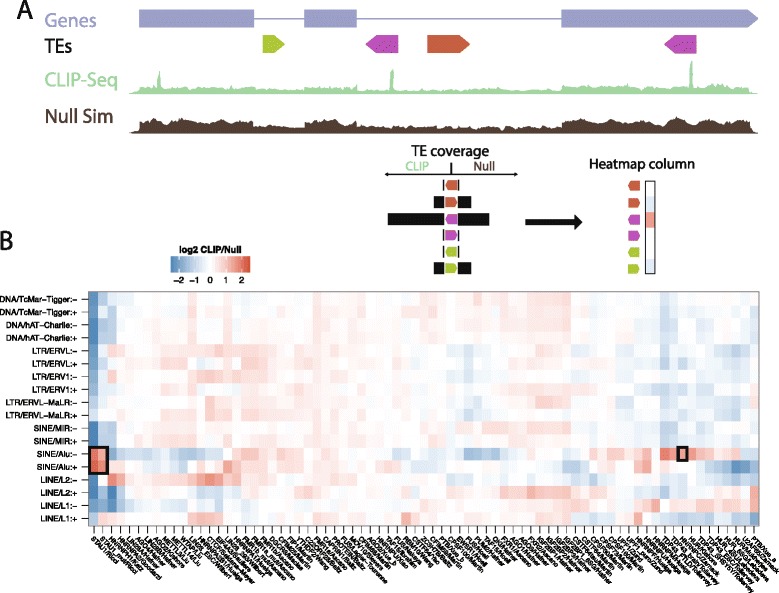
**CLIP-Seq alignments are enriched and depleted in specific TEs. (A)** Across the entire transcriptome (including introns), we counted overlapping aligned reads in the CLIP-Seq and null model simulation for every TE family in both orientations. The top track draws an example gene with blue boxes representing exons. The track below draws a set of example TEs, colored by family and with arrowhead describing orientation. Alignment coverage from CLIP-Seq and null simulation experiments are drawn below, with CLIP-Seq coverage spiking primarily on the purple TE in antisense orientation to the gene. **(B)** Heatmap in which we plotted the log2 ratio of the CLIP-Seq and null model counts, normalized by library size, for every pair of TE family in both orientations on the y-axis and RBP (with publication first author) on the x-axis. The heatmap was column mean normalized. Black rectangles highlight well-characterized interactions between STAU1 and Alu elements and hnRNP C and antisense Alu elements. TcMar-Tigger and hAT-Charlie are DNA transposons, which transpose via a cut-and-paste mechanism. ERVL, ERV1, and ERVL-MaLR are endogenous retrovirus families that have long terminal repeats (LTRs) on both ends. Both long interspersed nuclear elements (LINEs) and short interspersed nuclear elements (SINEs) are non-LTR retrotransposons, which mobilize via an RNA intermediate using a copy-and-paste mechanism.

To comprehensively survey RBP interactions with TEs in the human genome, we collected and systematically processed all accessible CLIP-Seq datasets, settling on 75 experiments mapping 51 RBPs (Materials and methods). First, we compared the number of aligned reads, relative to total library size, overlapping all instances of each TE family in both orientations in the CLIP-Seq to a null model expectation (Figure 1A). We can split each TE family into its sense and antisense orientations because CLIP-Seq uses a strand-specific library construction method. To control for differing mappability of the TE families and varying expression levels of transcripts containing specific TE families, we compared alignment coverage to simulated coverage from a null model (see Materials and methods).

We found many TE families were enriched for CLIP-Seq aligned reads from specific RBPs (Figure 1B). Enrichments and depletions of RBP-TE associations were broadly similar in mRNA exons, lncRNA exons, and introns (Figure S1 in Additional file 1). We benchmarked our approach by comparing it to previous CLIP studies discussing RBP-TE interactions. STAU1 binding to Alu sequence is a well-known phenomenon [20,34], and we confirmed that interaction here, observing a 3.2- to 4.1-fold enrichment of STAU1 alignments in Alu-derived sequence. Zarnack *et al*. [35] found that hnRNP C preferentially binds antisense Alu elements in RNA, where it prevents U2AF65 binding and aberrant splicing. Our analysis pipeline reproduced that interaction, detecting a 2.3-fold enrichment of hnRNP C alignments in antisense Alu elements throughout the transcriptome. TDP-43 binding to TE-derived sequence had also been previously noted [36]. We observed this phenomenon in the form of significant enrichments to antisense L1 (1.3- to 1.7-fold) and antisense Alu (1.5- to 2.3-fold) elements [37]. Together, these results demonstrate that our mapping and normalization pipeline confirms previously reported TE-driven RNA-protein interactions.

We further detected hundreds of significant novel enrichments between RBPs and TEs. The signal strength from even this low-resolution analysis — considering enrichment over the entirety of TE sequences - suggested that RBP-TE interactions are widespread. Thus, we proceeded to dissect these enrichments and their biological implications.

### CLIP-Seq alignments cluster on specific transposable element motifs

Having observed overall enrichment of alignments in families of TE-derived sequence, we next asked whether specific subregions of the repeat consensus sequences drive these enrichments. Many RBPs have specific sequence and/or structure preferences [16,38]. These preferred motifs may appear in TE sequence and manifest as peaks in alignment coverage on the TE consensus. To control for uneven mappability and genomic coverage of TEs, we compared CLIP-Seq alignment coverage to coverage from the uniform null model across TE consensus sequences (see Materials and methods).

**Figure 2:**
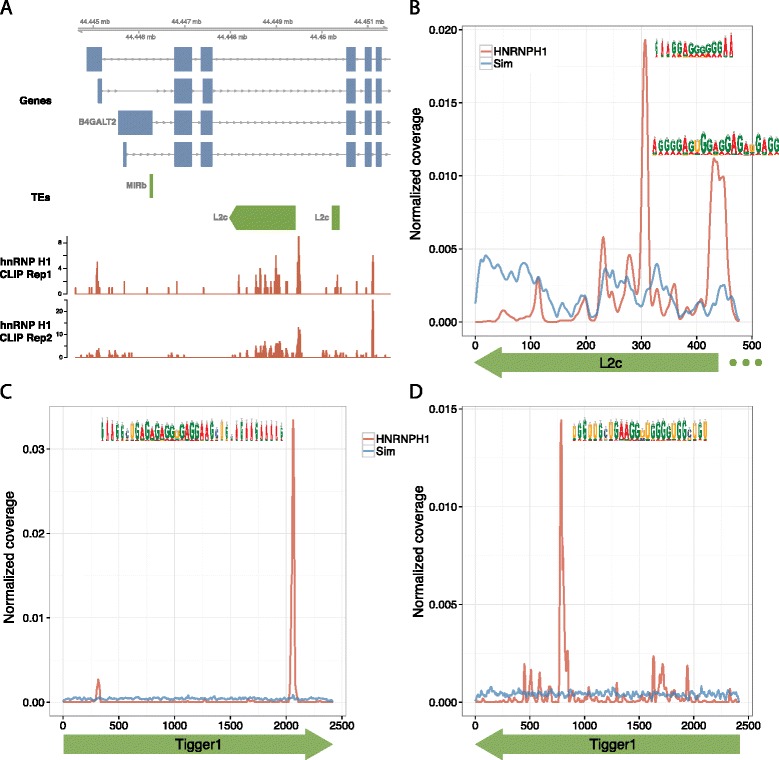
**hnRNP H1 CLIP-Seq coverage clusters at specific TE motifs. (A)** Two replicates of hnRNP H1 CLIP-Seq alignments show an alignment coverage peak at the same position of an antisense L2c element in *B4GALT2*. **(B)** hnRNP H1 alignments are enriched for antisense L2c overall, and coverage on the consensus 3′ end of the element clusters on two AG-rich positions. Coverage was normalized to sum to one across the TE span. **(C,D)** In contrast, Tigger1 is depleted for hnRNP H1 overall, but nearly all alignments cluster on particular positions in both the sense **(C)** and antisense orientation **(D)**.

Strikingly, both enriched RBP-TE pairs and many others without enrichment showed strong evidence of RBP binding to specific subregions within the TE consensus. For example, hnRNP H1 CLIP-Seq alignments clustered on two particular subregions of the antisense consensus of L2 elements (Figure 2A,B). Even sharper peaks appeared in both the sense and antisense orientations of the DNA transposon Tigger1 (Figure 2C,D), where the large size of the approximately 2,500-nucleotide element hid the interaction in Figure 1. In each case, the underlying sequence was AG-rich, in line with prior studies on hnRNP H1 binding preferences [39,40].

Having witnessed peaked coverage within TE subregions, we next compared and contrasted the preferences for these subregions among the many RBPs analyzed. In some cases, RBPs shared a preference - for example, a number of RBPs shared hnRNP C’s affinity for two poly-U tracts of antisense Alu elements, which are well-studied in their sense poly-A form (Figure S3 in Additional file 1) [41]. However, even for antisense Alu, we observed a diversity of binding profiles among enriched RBPs, with STAU1, FMR1, and hnRNP U preferring different subregions (Figure S3 in Additional file 1). RBPs sharing a similar binding profile in one TE did not generalize to common profiles in all TEs. For example, the splicing factor U2AF65 bound near hnRNP C in antisense Alu elements, where aberrant splicing is repressed [35], but the two RBPs bind apart in antisense L1 elements (Figure S4 in Additional file 1).

RBPs mapped using the same experimental protocol within a single study offer a valuable opportunity to ask whether the binding profiles are broadly similar between RBPs, which might occur if sequence composition biases of the protocol overwhelm the true signal. The differences between hnRNP C and U2AF65, both mapped by Zarnack *et al*. [35], in antisense L1 elements described above generalized to other major elements (Figure S4 in Additional file 1), lending credence to the authenticity of the detected interactions. As an additional example, CLIP-Seq HuR and Ago2 coverage profiles in Kishore *et al*. [42] diverge drastically (Figure S5 in Additional file 1).

Based on our observation that RBP alignment coverage clusters on specific subregions of TEs, we sought to systematically identify the underlying sequence motifs. To this end, we segmented coverage peaks where CLIP-Seq coverage was greater than three-fold more than the null model and refer to them hereafter as TE-specific motifs (TESMs). To standardize the TESMs for further analysis, we focused on nine nucleotide motifs centered at the maximum coverage nucleotide of each peak region. Although many known RBP motifs are shorter [16], we chose nine to include additional surrounding context and add specificity in studying the motif occurrences.

**Figure 3:**
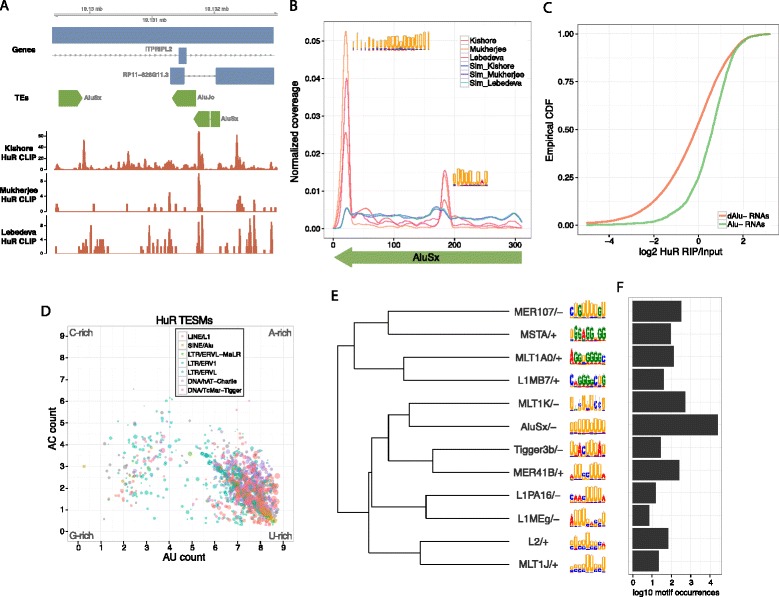
**HuR binds U-rich motifs in antisense Alu elements. (A)** CLIP-Seq alignments from three different studies demonstrated *HuR* binding within an antisense AluSx in the 3′ UTR of *ITPRIPL2*, **(B)** which generalized to genome-wide binding to two poly-U tracts in the antisense Alu consensus, shown here as alignment coverage normalized by dataset to sum to one across the element. **(C)** In a formaldehyde RNA immunoprecipitation (fRIP)-Seq of HuR, genes containing exonic antisense Alu elements (Alu- RNAs) were strongly enriched in the RIP over input compared with genes devoid of antisense Alu (dAlu- RNAs). Genes targeted via intronic antisense Alu elements also showed strong evidence of binding (Figure S6 in Additional file 1). **(D)** Plotting all HuR TE-specific motifs by their nucleotide composition revealed a diversity of motif compositions but a strong tendency towards Us. The x-axis specifies the expected number of As and Us in the motif model, and the y-axis specifies the expected number of As and Cs. Point size is proportional to the log2 of the number of CLIP-Seq alignments overlapped by the motif. **(E,F)** To better reveal the relationships between the strongest motifs, we collapsed highly redundant motifs into 12 representatives and hierarchically clustered them using information coverage Euclidean distance (see Materials and methods) [75].

The TESM sequences broadly matched the known binding preferences of the RBPs. In accordance with prior work, TE enrichments of the ubiquitously expressed stabilizing RBP HuR were driven by binding to U-rich regions (Figure 3) [43,44]. HuR appears to bind these U-rich motifs in prevalent antisense Alu, antisense L1, and sense L2 elements. Its affinity for antisense Alu focuses on the two poly-U tracts (Figure 3B).

Uridine is known to crosslink more efficiently in PAR-CLIP experiments [42,45], which was used by all three groups to map HuR [42-44]. To address the possible concern that this may produce false positives, especially in the prominent binding to U-rich antisense Alu-derived sequence, we performed an HuR formaldehyde crosslinked RNA immunoprecipitation and sequencing (fRIP-Seq), which does not suffer from a uridine bias (see Materials and methods). Consistent with the enriched CLIP-Seq alignment coverage, genes containing antisense Alu elements had far greater fold changes in our fRIP over input RNA than devoid genes (Figure 3C), which was true for both exonic and intronic occurrences of antisense Alu (Figure S6 in Additional file 1). In fact, antisense Alu elements were the strongest predictor of *HuR* binding among all TE families and orientations.

By leveraging the repetitive nature of TEs and combining CLIP-Seq signal over many copies of similar sequences, we extensively characterized the *in vivo* binding preferences of these RBPs. Plotting the nucleotide composition of HuR TESMs clearly shows their uridine richness, with a slight bend towards adenosines, matching prior expectations of HuR as a binder of AU-rich elements (Figure 3D) [46]. Surprisingly, we discovered multiple HuR TESMs with a different, somewhat G-rich composition (Figure 3E). These motifs only account for a small proportion of the alignments, but there is a strong enrichment for CLIP-Seq alignments at both their TE and nonrepetitive occurrences (Figure S7 in Additional file 1). Though the other HuR CLIP experiments offer a mixed view of the relevance of these sites (Figure S7 in Additional file 1), our HuR fRIP-Seq suggests their validity (Figure S8 in Additional file 1).

Altogether, we delineated 15,424 TESMs from the 75 datasets. The distribution of motif number varied widely between RBPs because the datasets differ in sequencing depth and enrichment of bound RNA over input (Figure S9 in Additional file 1). Clustering the datasets by their TESM coverage profiles revealed a diversity of RBP binding preferences, with a substantial group of AU-rich binders (Figure S9 in Additional file 1).

Overall, we found CLIP-Seq alignment coverage on TEs is highly nonuniform, clustering on thousands of TESMs, which generally matched the known RBP binding preferences but also uncover possible alternative binding modes. Enumerating and comparing the TESMs produces an extensive characterization of the *in vivo* binding preferences of the RBPs.

### Transposable element-specific RBP motifs are bound and conserved in the nonrepetitive transcriptome

We next compared the binding preferences of the RBPs within and outside of TEs. If a TESM is prevalent outside of the TE and attracts CLIP-Seq alignment coverage, it would serve to validate the RBP affinity for that motif. It might also suggest that the TEs, which are typically newer entrants into the genome, appropriated existing RBP sequence preferences.

For this analysis, we considered only the top 300 TESMs per dataset, which were chosen by collapsing highly redundant motifs (for example, from homologous positions of Alu subfamilies) and ranking by coverage enrichment over the null model (see Materials and methods). Across all datasets, we elucidated 5,546 TESMs with evidence of RBP binding. We mapped these motifs in the nonrepetitive portion of the transcriptome to study their properties.

**Figure 4:**
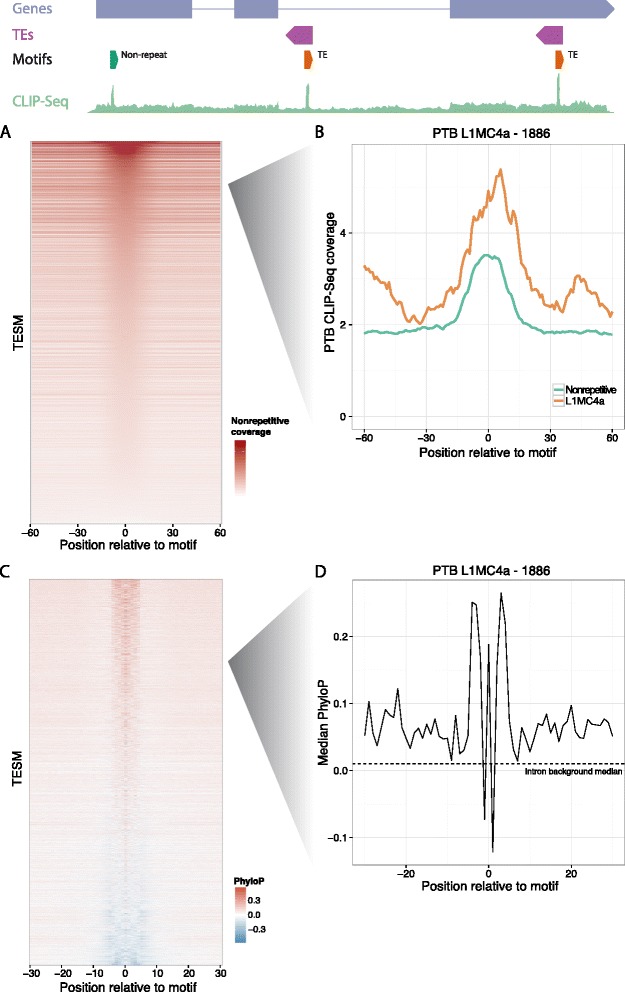
**TE-specific motifs are bound and conserved outside of TEs.** We mapped 5,546 TE-specific motifs around the nonrepetitive transcriptome. **(A)** CLIP-Seq alignment coverage indicated that most motifs were bound; 87% of motif instances showed increased coverage at the motif versus the surrounding 200 nucleotides. **(B)** A motif discovered in PTB CLIP-Seq on antisense L1MB2 exemplified this, with coverage both in and out of L1MC4a. **(C)** PhyloP conservation scores indicated that many motifs were also conserved. The heatmap plots the median PhyloP score across all intronic motif instances. **(D)** At finer resolution, mutation rates differed by position within the motif, exemplified here by a PTB antisense L1MC4a motif.

To assess CLIP-Seq coverage of the TESMs outside of repeats, we compared coverage directly at the motif with that in a surrounding 200 nucleotide region. We observed strong evidence that these motifs are bound outside of TE-derived sequences; 87% had increased coverage at the motif (Figure 4A), exemplified here by PTB CLIP-Seq coverage on nonrepetitive occurrences of a motif found in antisense L1MC4a (Figure 4B). Thus, RBP sequence preferences in TEs resemble those outside of repeats.

To further explore the potential for function in these nonrepetitive TESM occurrences, we considered their conservation using PhyloP (Figure 4C) [47]. Due to the severely different PhyloP backgrounds in the various annotation classes, we separated the analysis into introns, lncRNAs, and 3′ UTRs, ignoring coding sequence due to its much higher conservation signal. Seventy percent of motifs had a mean PhyloP above the intron baseline mean, exemplified again by an L1MC4a motif found for PTB (Figure 4D). The discovery of most motifs was driven by intronic sequencing coverage; accordingly, fewer motifs show constraint in the exonic sequence of 3′ UTRs (47%) and lncRNAs (45%). Nevertheless, for all annotation classes, TESM PhyloP distributions were significantly greater than expected by random sampling of 9-mers (Figure S10 in Additional file 1), despite the fact that 9-mers that appear in TE consensus sequences (approximately the set from which these TESMs were discovered) have a severely decreased PhyloP distribution overall (Figure S11 in Additional file 1). Motif conservation and CLIP-Seq coverage were not strongly related (Figure S12 in Additional file 1).

We next asked whether the PhyloP distributions differed by nucleotide position within a TESM. Indeed, plotting these distributions revealed nonuniform conservation of the motifs. Though in some cases constraint was present across the entire motif (Figure S13a in Additional file 1), in other cases only a subset of the nucleotides showed evidence of constraint in interesting and often symmetrical patterns (Figure S13b,c in Additional file 1). For a final set, we detected a high mutation rate across the motif (Figure S13d in Additional file 1), suggesting that many sequences throughout the nonrepetitive transcriptome have mutated towards these motifs.

Altogether, TESMs show evidence of binding to constrained sequences in the nonrepetitive transcriptome, suggesting that TEs have primarily appropriated the existing conserved binding preferences of these RBPs rather than binding through alternative mechanisms.

### Transposable element binding affects RNA abundance and splicing

The evidence that TEs have intercepted existing RBP binding preferences, coupled with the widespread binding of RBPs to TEs, begs the question of whether TE binding sites are functionally similar to nonrepetitive sites. To investigate this question, we collected RBP knockdown RNA-Seq experiments matching 12 of the analyzed CLIP-Seq datasets. These experiments can detect changes in the abundances and splicing of genes determined to be targeted by the RBP in the CLIP-Seq and were used in the original studies to understand the RBPs’ functions. We identified target genes with the CLIP-Seq using an enhanced version of established statistical procedures to call binding sites, considering both exons and introns, and computed differential expression between the paired RNA-Seq samples using Cuffdiff (see Materials and methods).

**Figure 5:**
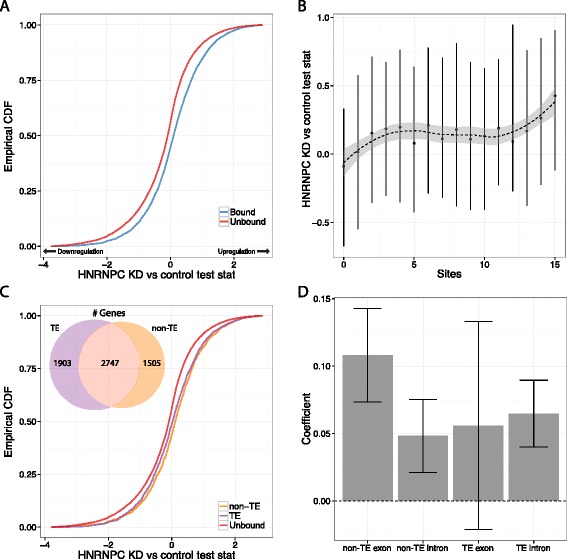
**hnRNP C-TE binding sites repress genes similarly to nonrepetitive sites. (A)** Genes targeted by hnRNP C were upregulated after hnRNP C knockdown (KD) compared to unbound genes, shown here as the cumulative distributions of the Cuffdiff differential expression test statistic (CDF). Positive values indicated greater abundance in the knockdown. **(B)** The test statistic distribution, plotted as the median and interquartile range, increased with the number of binding sites identified in the gene span. **(C)** Genes targeted only in TE sites were upregulated similarly to genes targeted only in nonrepetitive sites. The Venn diagram indicates the wide scale of TE binding sites, depicting the number of genes bound only in TEs, only in non-TEs, and in both. **(D)** A linear regression on the logarithm of the number of sites in each class showed that upregulation had a positive relationship with site number for all categories. Error bars represent a 95% confidence interval. The TE exon coefficient has large error bars because there were few examples to learn from.

We first asked whether hnRNP C knockdown impacted TE binding sites similarly to nonrepetitive binding sites. Target genes tended to be upregulated after RNA interference-mediated depletion of hnRNP C (Figure 5A), suggesting a destabilizing effect on bound transcripts that increases with the number of binding sites (Figure 5B). To separately measure the effect of TE and nonrepetitive binding sites, we plotted the cumulative distributions of Cuffdiff’s differential expression test statistic for genes bound only in nonrepetitive sequence or only in TEs (Figure 5C). As hypothesized, genes bound only in nonrepetitive sequence and only in TEs were similarly upregulated. This result held up separately for mRNAs and lncRNAs (Figure S14 in Additional file 1).

To better understand the effect of the various types of binding sites in genes targeted at multiple sites, we computed a linear regression to predict the differential expression test statistic using the logarithms of the number of binding sites in each class, further divided by exon and intron. The positive model coefficients augment the case that TE-derived hnRNP C binding sites repress target genes to a similar magnitude as nonrepetitive sites (Figure 5D).

To determine if TE-derived binding sites affect alternative splicing, we examined Cuffdiff *P*-values for the Materials and methodsstatistical significance of an isoform switch (see Materials and methods). Misregulation of splicing in antisense Alu elements was the primary phenotype described for hnRNP C in these data [35]; accordingly, we found that genes bound by hnRNP C had lesser splicing difference *P*-values, indicating more alternative splicing, after hnRNP C knockdown (Figure S15a in Additional file 1). Further, genes with more sites had more evidence for splicing differences (Figure S15b in Additional file 1). Binding sites in TEs affected splicing of their genes similarly to nonrepetitive sites (Figure S15c,d in Additional file 1). Alu and non-Alu TE sites were indistinguishable, suggesting the novel insight that hnRNP C’s function as a splicing repressor generalizes beyond Alu elements.

**Figure 6:**
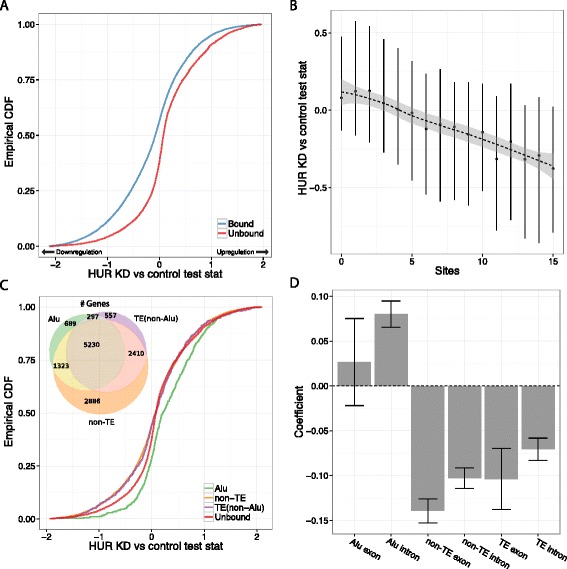
**HuR-TE binding sites stabilize genes, unless in Alu elements. (A)** As expected for the known transcript stabilizer HuR, targeted genes were downregulated after HuR knockdown (KD) compared with unbound genes, shown here as the cumulative distributions of the Cuffdiff differential expression test statistic (CDF). **(B)** HuR’s stabilizing effect depended strongly on the number of binding sites in the gene span, shown through the decreasing medians and interquartile ranges of the test statistic distribution. **(C)** Unexpectedly, binding sites in Alu elements had the opposite effect; genes targeted only in Alu elements were upregulated after HuR knockdown. All other TE binding sites had the expected effect with similar magnitude as nonrepetitive sites. The Venn diagram indicates the wide scale of TE binding sites, depicting the number of genes bound only in Alu elements, only in non-Alu TEs, only in non-TEs, and in various mixtures. **(D)** A linear regression on the logarithm of the number of sites in each class verified that non-Alu TE and nonrepetitive sites predicted downregulation, but Alu sites did not. Error bars represent a 95% confidence interval. The Alu exon and TE exon coefficients have large error bars because there were few examples to learn from.

We next examined the effect of HuR depletion on genes with TE-derived binding sites in RNA-Seq experiments from two studies [42,44]. Both found that HuR stabilized target genes, as genes targeted in the CLIP-Seq were significantly downregulated upon HuR knockdown. We reproduced these results but focused further analysis on the Kishore *et al*. dataset because bound genes were more affected by the knockdown (Figure 6A; Figure S16 in Additional file 1).

HuR target sites in TEs generally function similarly to nonrepetitive sites, but depend on the family bound. Genes targeted via non-Alu TEs were similarly downregulated after HuR knockdown (Figure 6C). Surprisingly, downregulation was absent for genes targeted only in Alu elements, which tended to change less than unbound genes in both directions (Figure 6C). These effects were apparent in both mRNAs and lncRNAs separately (Figure S14 in Additional file 1). As above, we computed a linear regression to quantify the effect of binding sites in these various annotation classes. The opposing model coefficients furthered the case that non-Alu TE-derived HuR binding sites stabilize the gene, but Alu binding sites do not (Figure 6D,E).

The remaining knockdown experiments further corroborated the significant effect of TE binding sites. Binding to TE-derived sites by both hnRNP H1 (Figure S17 in Additional file 1) and hnRNP U (Figure S18 in Additional file 1) stabilizes transcripts, while TE binding by PTB (Figure S19 in Additional file 1), WTAP (Figure S20 in Additional file 1), and METTL3 (Figure S21 in Additional file 1) represses transcripts. METTL3 serves as an additional example where activity depends on the TE family bound; L1 sites buck the general trend and appear to stabilize the transcript (Figure S21 in Additional file 1). Splicing analysis for most experiments was underpowered by having performed only single replicates, but METTL3 also showed a phenotype, with TE-derived sites increasing the likelihood of an isoform switch after knockdown (Figure S21 in Additional file 1).

In summary, RBP knockdown gene expression analyses establish that TE-derived and nonrepetitive RBP binding sites affect RNA state similarly, with interesting counterexamples, like Alu-HuR interactions, where the TE binding context may alter function. Extrapolating these results, the thousands of RBP-TE binding sites discovered in this analysis are candidates for function via RNA-protein interaction.

## Discussion

Recent research has described a substantial role for TEs in the evolution of gene regulation at the transcriptional level; for example, TEs have dispersed transcriptional regulatory signals around the genome, and many sites have been co-opted for essential functions [7]. However, the influence of TEs on post-transcriptional regulation has previously been limited to a few promising examples. Here, we globally and systematically studied binding of RBPs to TE-derived sequence in human RNAs using a diverse set of CLIP-Seq experiments. We discovered widespread enrichment of RBPs on individual TE families, driven by sequence composition preferences of the RBPs for specific regions of those TEs. We described and studied thousands of these TESMs.

Many RBPs preferred U-rich TESMs, which was of notable concern because uridine is known to crosslink more efficiently in some CLIP experiments [42,45]. In most cases, the RBPs preference for U-rich sequence was previously known, such as HuR, hnRNP C, FUS, among others. But to more definitively test the many U-rich interactions of HuR, we performed a fRIP-Seq, which does not suffer from more efficient crosslinking of uridines. The experiment validated HuR interaction with many U-rich sequences like antisense Alu elements as major contributors to HuR binding.

We accumulated compelling evidence that TESMs are relevant not only within TEs but also in the nonrepetitive transcriptome. For most TESMs, CLIP-Seq alignment coverage increased over motif occurrences outside of repeats. These motifs also showed greater than expected conservation in nonrepetitive 3′ UTR, intron, and lncRNA sequences. Together, these observations suggest that the RBPs’ sequence preferences for these motifs were already established, and TE-derived instances of the motifs intercepted these preferences upon entry into the genome.

Despite this potentially ‘uninvited’ entry into post-transcriptional regulatory networks, we found that most RBP-TE binding sites affect RNA state with the same effect and to a similar magnitude as binding sites in nonrepetitive sequence. In addition to reproducing the impact of hnRNP C binding to antisense Alu elements on splicing [35], we discovered that many more hnRNP C binding sites on other TE families also affect splicing and transcript abundance upon hnRNP C depletion. Depletion of additional RBPs (hnRNP H1, hnRNP U, PTB, WTAP, and METTL3) introduced more evidence for functional TE binding as genes targeted via TE-derived sites had similarly altered abundance to genes targeted via nonrepetitive sites. Thus, we have gathered here considerable evidence that often-ignored TE binding sites should be considered alongside nonrepetitive sites for their potential to modulate RNA abundance and splicing.

We also observed cases of TE-dependent regulation, such as HuR binding to antisense Alu elements. HuR binding sites stabilize the RNA such that HuR depletion causes downregulation of target genes. While we observed this influence for most TE-derived binding sites, the many Alu-derived sites were a conspicuous exception. Alu-bound genes were both less downregulated and less upregulated than unbound genes, suggesting that the abundance level of these genes is resistant to change in either direction. Most likely, we are missing the full picture at these Alu sites and combinatorial binding of multiple RBPs determine the effect on abundance. Follow-up experiments are needed to unravel this complex case.

The evidence here that RBP binding to TEs is widespread and can produce measurable and sometimes complex effects on gene abundance and splicing begs the question of how disrupting these interactions might affect the health of cells and organisms. RBPs have been implicated in numerous genetic diseases [48], including recent associations with neurodegenerative disorders [49] such as amyotrophic lateral sclerosis [50]. TEs, too, are a substantial focus of disease research, primarily with respect to deleterious novel transposition events [51,52], but also via misregulation of TE RNA [53]. Disruption of RBP-TE interaction homeostasis via RBP mutations or TE misregulation is a new and important avenue to consider in the etiology of human disease.

A considerable proportion of TEs in the exonic transcriptome lies in lncRNAs [24,25]. The myriad lncRNAs implicated for critical roles in development [54-56] and disease [57,58] emphasize the need for improved understanding of lncRNA function. A modular domain structure for lncRNAs has been hypothesized [59] but has, thus far, eluded a thorough characterization. Our observation that TE sequences contain functional RBP binding sites represents an important step towards characterizing TEs as one type of modular domain in lncRNAs where RBP-TE interactions may function.

Alterations to transcriptional regulatory networks are a major driver of evolutionary change [60,61]. TEs containing transcription factor binding sites play a substantial role in creating the variation that provides the raw material for selection to operate on [9,10,24,62]. Comparisons across species show that lineage-specific TEs can rapidly rewire regulatory networks [11-13,63]. Ultimately, these transcriptional regulatory site changes drive morphological change because they modulate protein abundance. Through a variety of mechanisms acting on RNA, such as splicing, localization, and degradation, post-transcriptional regulation by RBPs also modulates protein abundance. Our observation that RBP binding to TEs is widespread and can produce measurable effects on gene abundance and splicing suggests that TEs may also provide variation for post-transcriptional regulatory evolution. Mapping RBP-TE interactions in more species and placing them in the context of development and the adaptive responses of adult cells will elucidate the degree to which these interactions, too, are a major driver of evolutionary change.

## Materials and methods

### CLIP-Seq data and processing

We downloaded 75 CLIP-Seq datasets from 31 studies mapping 51 RBPs from the Gene Expression Omnibus (GEO), Sequence Read Archive, and EMBL ArrayExpress.

CLIP-Seq experiments typically build sequencing libraries using a small RNA protocol, which attaches a sequence adapter to the suffix of the short read. Unfortunately, these adapters are rarely reported in the manuscript or public database entry. Rather than take on the impractical, and perhaps impossible, task of acquiring accurate adapter information for 75 datasets, we implemented an adapter-ignorant strategy to align the prefixes of these reads up to the putative adapter sequence.

We aligned with TopHat 2.0.9 [64] to human genome assembly hg19, providing GENCODE v18 as reference annotation [65]. To align read prefixes, we carried out the following steps. First, we attempted to align the first 20 nucleotides of the read. For every read, if a unique alignment was found, we returned that alignment. If multiple alignments were found, we added it back to a set for re-alignment with an additional nucleotide added back to the end of the read prefix. We repeated this procedure, re-aligning ambiguous read prefixes up to the full read length. When a read that aligned in one iteration fails to align in the next, we presumably encountered the adapter and returned the read’s alignment(s) from the previous iteration. Open source Python code implementing this strategy is available from [66].

The primary error that this pipeline can make is to distribute a highly repetitive read in a biased manner to genomic positions where the nucleotides downstream of the true read match the prefix of the adapter by chance. This error is not problematic for the analyses here where we merely need reads from TE-derived sequence to be aligned to some instance(s) of the TE family.

Due to low input material, CLIP-Seq experiments tend to have many PCR duplicated reads. We found allowing two alignments per chromosomal position struck a good balance between throwing away misleading PCR duplicates and keeping informative alignments from highly expressed genes and enriched clusters where redundancy is expected. Finally, we merged replicate experiments into one alignment file.

After alignment and filtering duplicates, numerous datasets contained so few reads that their reliability for the downstream analyses was questionable. Thus, we removed any dataset containing <200,000 aligned reads.

### Multi-mapping reads

Careful interpretation of multi-mapping reads is critical to studying repetitive TE-derived sequences. We output 20 alignments per read with TopHat, which was found in a ChIP-Seq analysis of multi-mapping reads to be approximately the point where accuracy levels off [67]. That is, for reads with greater than 20 alignments, 20 are randomly chosen to be output. In all counting analyses described, we normalized alignments by the number of alignments of the read to account for the uncertainty of the true source alignment. For example, a read with 20 alignments will count 1/20 at each aligned position. As mentioned above, we note that most analyses performed only require that the read aligned to any instance of a TE family, which may or may not be the true source instance of the read.

### Transposable element alignment enrichment/depletion

We computed the number of CLIP-Seq reads in each dataset that overlap each TE family from RepeatMasker [68] in both orientations using BEDTools [69] and compared the counts with a null model that accounts for the differing abundances of transcripts and assumes uniform coverage along those transcripts. CLIP-Seq experiments typically have substantial background read alignments, which allowed us to approximate these abundance estimates by running Cufflinks [70] on the CLIP-Seq alignments themselves, using the --multi-read-correct option to more accurately distribute multi-mapping reads. In order to account for introns, we augmented the GENCODE v18 annotation with unspliced pre-RNA isoforms [65]. Using these abundance estimates, we simulated new reads uniformly along the transcripts and mapped these reads back to the genome. We computed enrichment/depletion as the log ratio of the proportion of reads in the true and null model datasets overlapping each TE family in both orientations.

### Transposable element consensus alignment coverage

To plot read coverage along the consensus sequence for each RBP-TE pair, we aligned all reads overlapping each TE family to its DFAM profile hidden Markov model [71] using HMMer [72]. To adjust for the influence of both mappability and the nonuniform presence of the TE consensus (for example, genomic instances of LINE1 often include only the 3′ end [73]), we normalized the actual read coverage by coverage from the null model simulated reads described above.

### Transposable element-specific motifs

We characterized sequence motifs underlying the alignment coverage peaks by identifying regions of the TE consensus profile hidden Markov model for which CLIP-Seq alignment coverage was more than three-fold greater than the null simulation alignment coverage. For each of these coverage peaks, we represented the motif as a position weight matrix, with column frequencies defined by the multiple sequence alignment of aligned reads. We primarily studied nine nucleotide motifs, centered at the maximum alignment coverage nucleotide of each peak. We mapped motifs throughout the transcriptome using PoSSuM and *P*-value threshold 1e-5 [74].

### Transposable element-specific motif clustering

At multiple stages of the motif analysis, we wanted to better understand the relationship between motifs and collapse highly similar motifs (for example, from homologous positions of Alu subfamilies) to avoid redundant computation. We chose information coverage Euclidean distance, an effective distance computed on position weight matrices, to quantify motif similarity [75]. Information coverage measures how informative a column in the position weight matrix is. For example, a uniform distribution of the four nucleotides would have zero information, and a column with only one valid nucleotide would have maximal information. Given two position weight matrices, we find their ungapped alignment with the minimum sum of Euclidean distances between column nucleotide distributions, weighted by the columns’ information coverages. That is, we more strongly consider similar nucleotide distributions at informative over uninformative columns.

In our analysis of the full set of TESMs across datasets, we collapsed motifs within each dataset by computing pairwise distances as above and performing an average linkage hierarchical clustering, flattening the clusters at a threshold of 0.15. To form the final set, we chose the top 300 TESMs per dataset after ranking by coverage enrichment over the null model.

### HuR formaldehyde RIP-Seq

#### Cell culture and cross-linking

K562 cells (ATCC catalog number CCL-243) were grown in RPMI 1640 (Invitrogen; Carlsbad, CA USA; catalog number 22400105) with 10% fetal bovine serum and 1% Antibiotic-Antimycotic 100X (Invitrogen; Carlsbad, CA USA; catalog number 15240062). We collected cells with a gentle 5 minute spin (500 g) and washed them with room temperature phosphate-buffered saline. We re-suspended at 5e6 cells per ml in room temperature RPMI media sans fetal bovine serum or Antibiotic-Antimycotic and added formaldehyde to a final concentration of 0.1%. We crosslinked at room temperature for 10 minutes and then halted it by quenching for 5 minutes at room temperature after adding glycine to a final concentration of 125 mM at a medium pace. We spun cells for 5 minutes at 500 g and washed twice in 4°C phosphate-buffered saline. We flash froze pellets of 10e6 cells and stored them at -80°C.

#### fRIP

We re-suspended frozen pellets in 1 ml of RIPA lysis buffer (50 mM Tris (pH 8), 150 mM KCl, 0.1% SDS, 1% Triton-X, 5 mM EDTA, 0.5% sodium deoxycholate, 0.5 mM dithiothreitol (add fresh) plus protease inhibitor cocktail (Thermo Scientific; Waltham, MA, USA; PI-87785) plus 100 U/ml RNaseOUT™ (Life Technologies; Woburn, MA, USA; catalog number 10777-019). We incubated cells at 4°C for 10 minutes before lysing on a Branson® digital sonifier (Emerson Industrial Automation; St. Louis, MO, USA) using 10% amplitude for 0.7 s on and 1.3 s off at 30 s intervals for a total of 90 s. We used chilled tube holders and swapped them out between shearing runs to reduce temperature elevation. After lysis, we spun the lysate at 4°C at maximum speed for 10 minutes. We collected supernatant and diluted by adding equal volume of fRIP binding/wash buffer (150 mM KCl, 25 mM Tris (pH 7.5), 5 mM EDTA, 0.5% NP-40, 0.5 mM DTT (add fresh), 1× protease inhibitor cocktail (add fresh), 100 U/mL RNaseOUT (add fresh)). At this point, we removed 50 μl of lysate for input sample and stored it at -20°C for later RNA purification and library construction. After dilution, we clarified the lysate by passage through a 0.45 μM syringe filter. We then ‘pre-cleared’ filtered lysate by incubating with Dynabeads® Protein G (Life Technologies; Woburn, MA, USA; catalog number 10004D) at a concentration of 25 μl of beads per 5 million cells for 30 minutes at 4°C with slow rotation. We flash froze pre-cleared lysate in 1 ml aliquots of approximately 5 million cells and stored it at -80°C. For fRIP, we thawed lysate on ice and added 6 μg of HuR antibody (Santa Cruz Biotechnology; Dallas, TX, USA; catalog number sc-5483). After addition of antibody, we rotated lysate at 4°C for 2 h before adding 50 μl of Dynabeads® Protein G. We rotated beads and lysate at 4°C for 1 h before washing twice with 1 ml of fRIP binding/washing buffer plus 1× protease inhibitor cocktail and 100 U/mL RNaseOUT. After the final wash, we removed the supernatant and froze and stored the beads at -20°C.

#### RNA purification and library construction

We resuspended the frozen beads in 56 μl of RNase-free water and added 33 μl of 3× reverse-crosslinking buffer (3× phosphate-buffered saline (without Mg or Ca), 6% N-lauroyl sarcosine, 30 mM EDTA, 15 mM dithiothreitol (add fresh)), 10 μl of Proteinase K (Life Technologies; Woburn, MA, USA; catalog number AM9516), and 1 μl of RNaseOUT to both the re-suspended beads and input sample. We performed protein degradation and reverse-crosslinking for 1 h at 42°C, then another 1 h at 55°C. We added beads and reaction buffer to 1 ml of TriZol (Life Technologies; Woburn, MA, USA; catalog number 15596-026). After agitation, we added 200 μl of chloroform followed by approximately 15 s of vigorous agitation and a 20 minute microcentrifuge spin at 4°C at maximum speed. We collected the aqueous layer, added it to 750 μl of ethanol plus 1 μl GlycoBlue™, and ran it over a Qiagen RNeasy® min-elute column (Qiagen; Valencia, CA, USA; catalog number 74204). We extracted RNA using the buffer RWT/3X isopropanol modification detailed in ‘Appendix B: Optional On-Column DNAse Digestion…’ of the Qiagen miRNeasy® Mini Handbook. We eluted RNA in 15 μl of RNase-free water. To remove ribosomal RNA, we fed ≥70 ng of input and fRIP RNA into the Ribo-Zero™ Magnetic Gold Kit (Epicentre; Madison, WI, USA; catalog number MRZG12324) followed by a cleanup using Agencourt RNAClean XP beads (Beckman Coulter; Brea, CA, USA; catalog number A63987) and elution with 19.5 μl of Elute, Prime, Fragment mix from the TruSeq RNA Sample Preparation Kit (Illumina; San Diego, CA, USA; catalog number RS-122-2001). We performed library construction per the vendor’s instructions, starting with the ‘Incubate RFP’ step. We pooled the resulting cDNA libraries and subjected them to paired-end sequencing on an Illumina HiSeq 2500 at a depth of 31 base pairs per read.

#### Computational analysis

We aligned fRIP-Seq reads to hg19 and GENCODE v18 reference annotation using TopHat 2.0.9 [64] and ran Cuffdiff 2.1.1 [76] to estimate gene abundances and perform statistical comparisons between the fRIP versus input alignments. Raw reads and Cuffdiff output have been deposited in GEO as record GSE61238.

### CLIP-Seq peak calling

To study the impact of RBP knockdown, we needed to define bound and unbound genes. We did so by annotating binding sites from the CLIP-Seq alignment coverage using a method based on prior CLIP-Seq scan statistic-based peak calling strategies [77], but with enhanced modeling of the multi-isoform structure of most human genes. A software implementation is available at [66].

Our peak calling strategy proceeded as follows. To avoid false positive peak calls from the very frequent PCR duplications without grossly betraying the scan statistic model assumptions (that is, that duplicate reads occur naturally), we first capped the number of reads aligning to the same chromosome and position at two. Next, to parameterize the scan statistic model, we estimated isoform abundances using Cufflinks and the --multi-read-correct and --compatible-hits-norm options. As described above, we augmented the reference transcriptome with unspliced pre-RNA isoforms in order to capture intron binding sites. We computed enriched 30-nucleotide windows using a Poisson scan statistic approach [78], where each window was parameterized based on the abundances of the overlapping isoforms. We weighted multi-mapping read alignments by the inverse of their read’s total number of alignments. Merging enriched windows produced the final peak calls. This procedure can be framed as an isoform-aware version of the Poisson-based methods commonly used for CLIP-Seq peak calling [77]. Finally, to focus this analysis on high confidence RBP targets, we removed peak calls overlapping particularly challenging genomic regions using a precomputed index, similar to the Genome Mappability Score [79], but computed with TopHat.

### Knockdown differential expression

We determined differentially expressed genes after RBP knockdown by aligning RNA-Seq reads to hg19 and GENCODE v18 reference annotation using TopHat 2.0.9 [64] and running Cuffdiff 2.1.1 [76] to compare RNA-Seq alignments. Because most experiments were performed as only single replicates, and were thus underpowered to detect significant changes, we primarily studied the differential expression test statistic assigned to every gene, which quantifies the significance of the observed change in the number of fragments per kilobase per million reads (FPKM).

Cuffdiff analyzes differential splicing by computing the Jensen-Shannon metric between the two conditions’ distributions of FPKM among the multiple isoforms from a transcription start site. Again, due to underpowered experiments, we primarily studied the *P*-values assigned to each gene transcription start site, which measure the significance of the observed splicing difference.

### Visualization

Genome browser figures were constructed with GViz [80] or IGV [81].

### Data availability

All CLIP-Seq datasets are publicly available with accession numbers specified in Additional file 2. HuR fRIP-Seq reads and Cuffdiff output are available as record GSE61238 in GEO.

## Additional files

Additional file 1:
**Supplementary figures.**
Additional file 2:
**CLIP-Seq dataset descriptions and public database accessions.**

## Abbreviations

CLIP: crosslinked immunoprecipitation
fRIP: formaldehyde RNA immunoprecipitation
GEO: Gene Expression Omnibus
lncRNA: long noncoding RNA
PCR: polymerase chain reaction
RBP: RNA binding protein
TE: transposable element
TESM: transposable element-specific motif
UTR: untranslated region
