# Supplementary material for: Transposable elements modulate human RNA abundance and splicing via specific RNA-protein interactions
Source: Genome Biol. 2014 Dec 3;15(12):537. doi: 10.1186/s13059-014-0537-5 (PMC4272801; doi:10.1186/s13059-014-0537-5)
Supplement: Additional file 1: — Supplementary figures. [file 13059_2014_537_MOESM1_ESM.pdf]

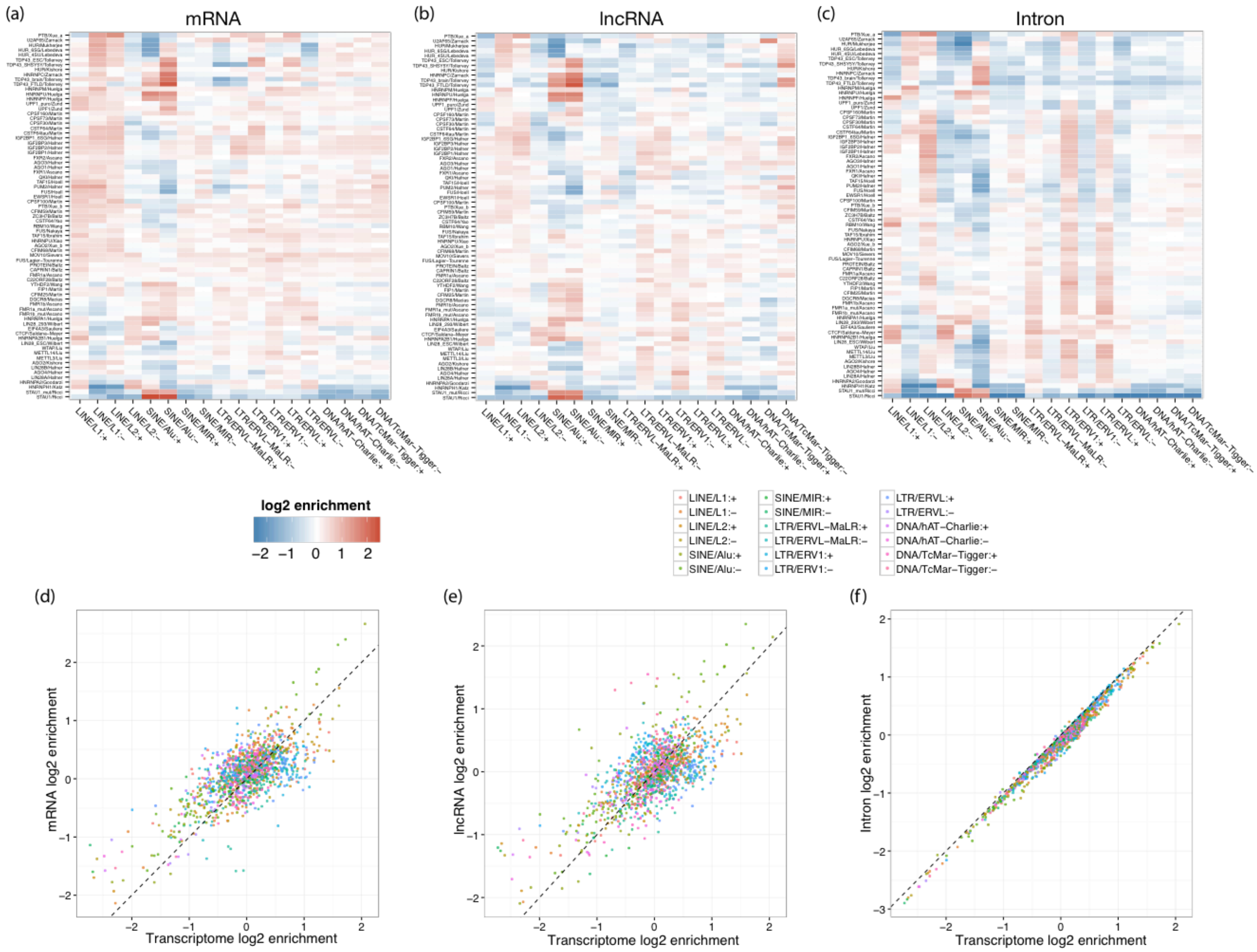

Supplementary Figure 1: CLIP-Seq enrichments/depletions are broadly similar in various annotation classes.

Using the same procedure as in Figure 1b, we computed enrichment in (a) mRNA exons, (b) lncRNA exons, and (c) introns independently. We further plotted the full transcriptome enrichment versus (d) mRNA, (e) lncRNA, and (f) intron enrichment. Note that enrichment in sense strand ERVs is poorly estimated due to incomplete annotation of isolated transcribed copies (Supplementary Figure 2).

(a)

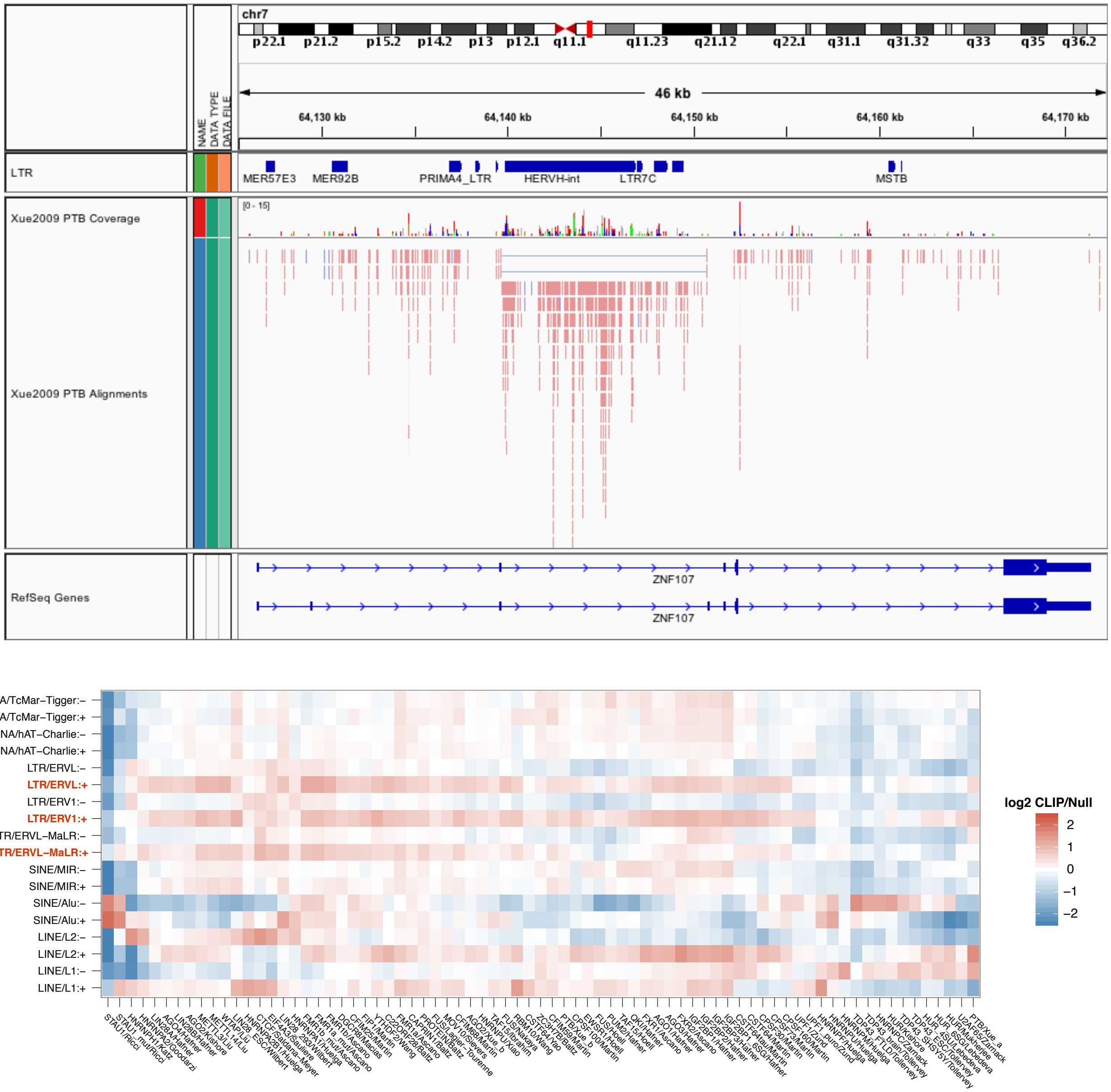

Supplementary Figure 2: Sense strand ERVs are enriched due to incomplete annotation. (a) Many isolated transcribed copies of endogenous retroviruses (ERVs) that are missing from the GENCODE v18 annotation, such as the one shown here in an intron of ZNF107, cause misestimation of the transcript abundance for the local gene's isoforms. (b) This biases the null model and tends to cause enrichment of sense strand ERVs.

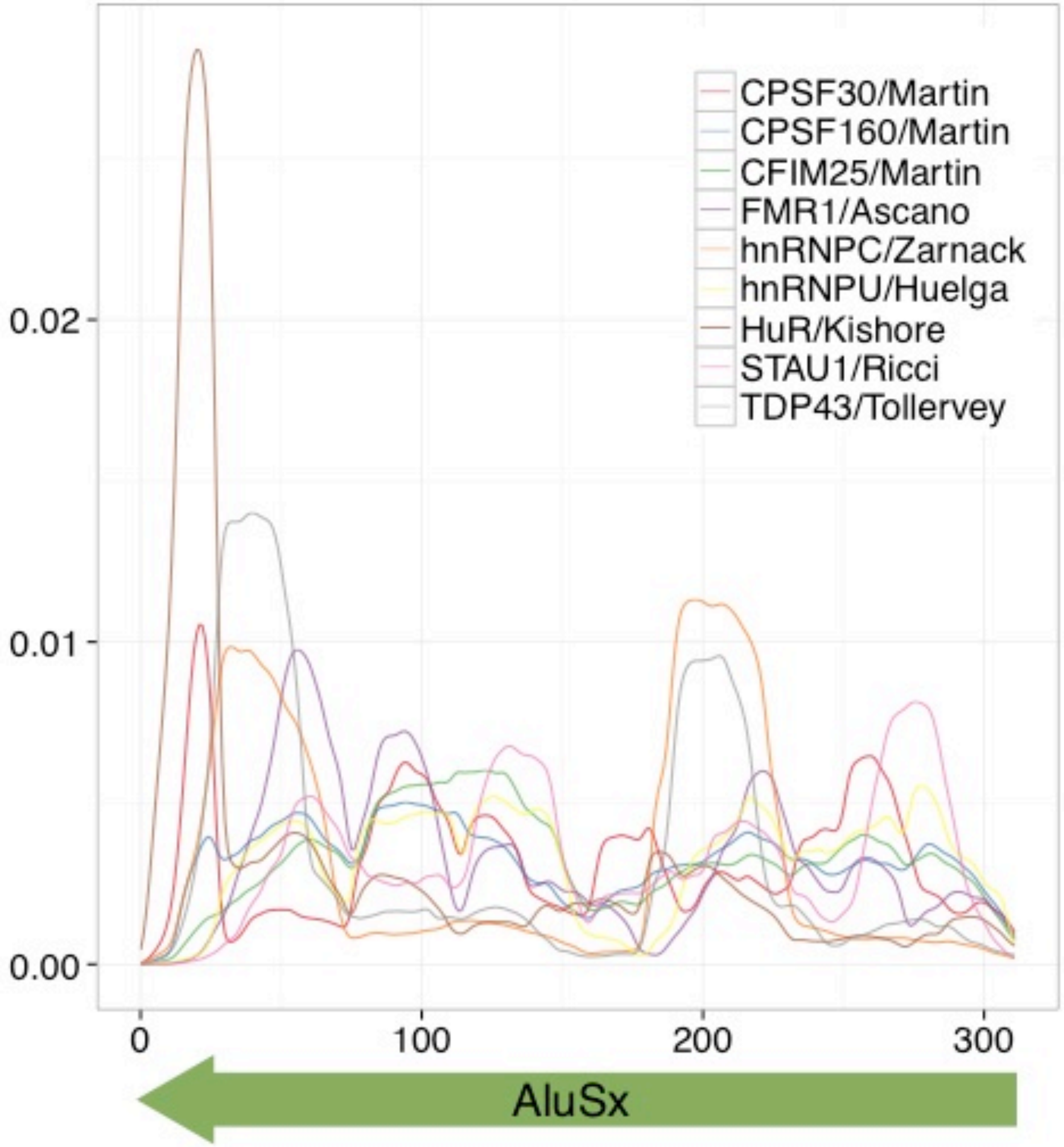

Supplementary Figure 3: Many, but not all, RBPs enriched for antisense Alu bind its U-tracts.

We plotted CLIP-Seq alignment coverage on antisense AluSx for all RBPs enriched >1.8 fold. Many RBPs bind the U-tracts at indexes ~20 and ~200. Others like *FMR1*, *CBSF160*, *CFIM25, hnRNP U, and STAU1* cluster at alternative regions.

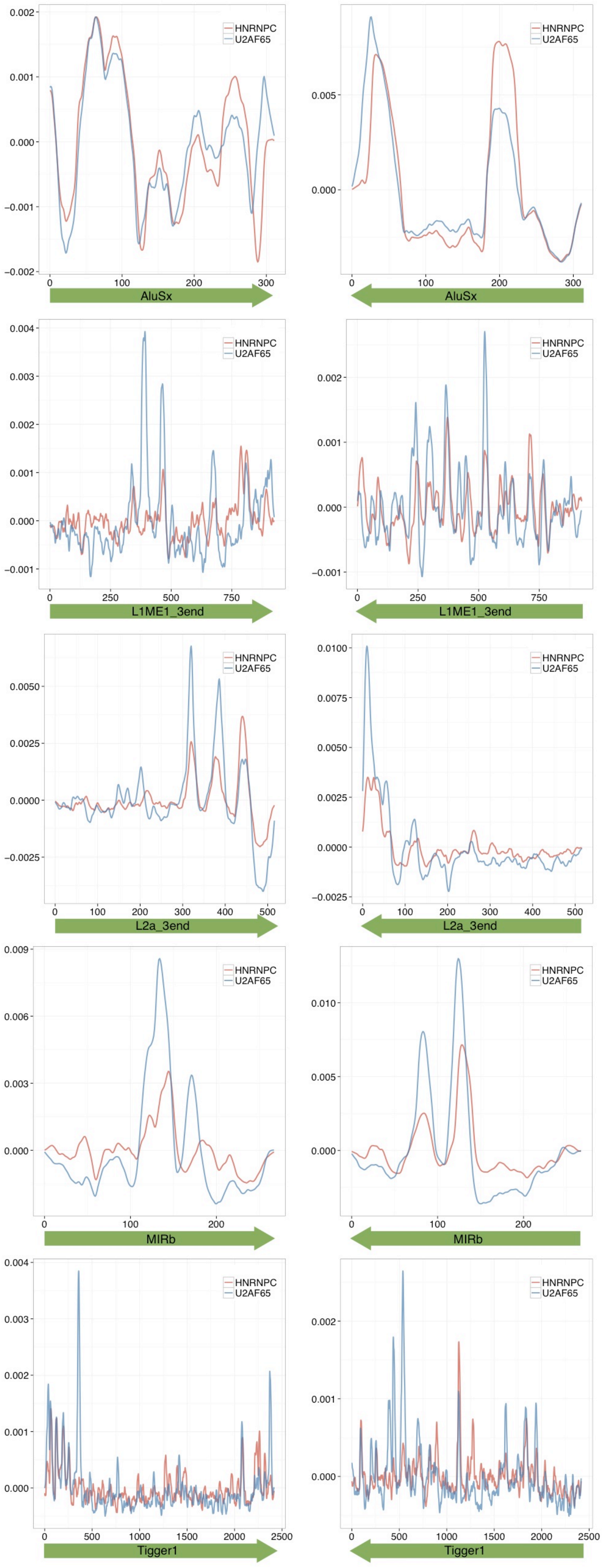

Supplementary Figure 4: Zarnack et al. *hnRNP C* and *U2AF65* CLIP-Seq alignments cluster at some similar and some different sites throughout major TE families.
Both performed in the same study by Zarnack et al., *hnRNP C* and *U2AF65* CLIP-Seq alignment coverage peaks at similar sites in some major TE families, such as antisense Alu elements, but different sites in others, such as sense L1ME1 elements.

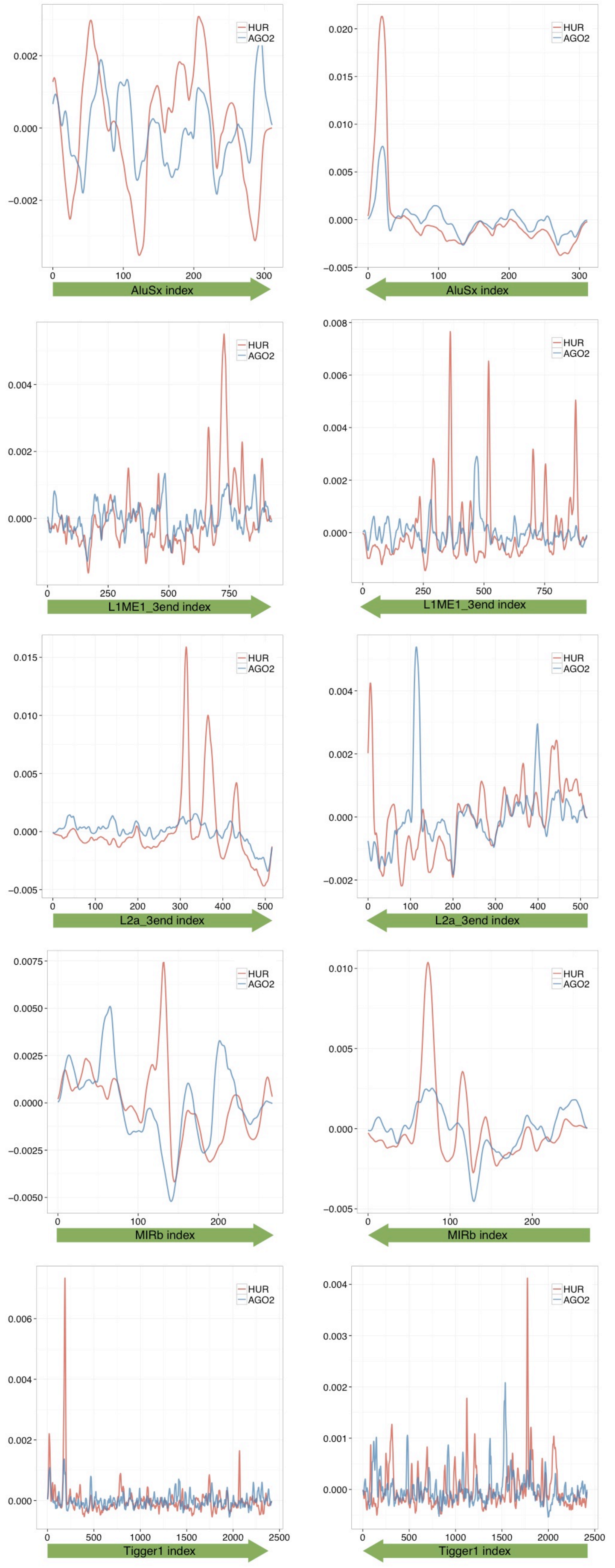

Supplementary Figure 5: Kishore et al. *HuR* and *AGO2* CLIP-Seq alignments cluster at different sites throughout major TE families. Both performed in the same study by Kishore et al., *hnRNP C* and *U2AF65* CLIP-Seq alignment coverage peaks at almost entirely different sites in some major TE families.

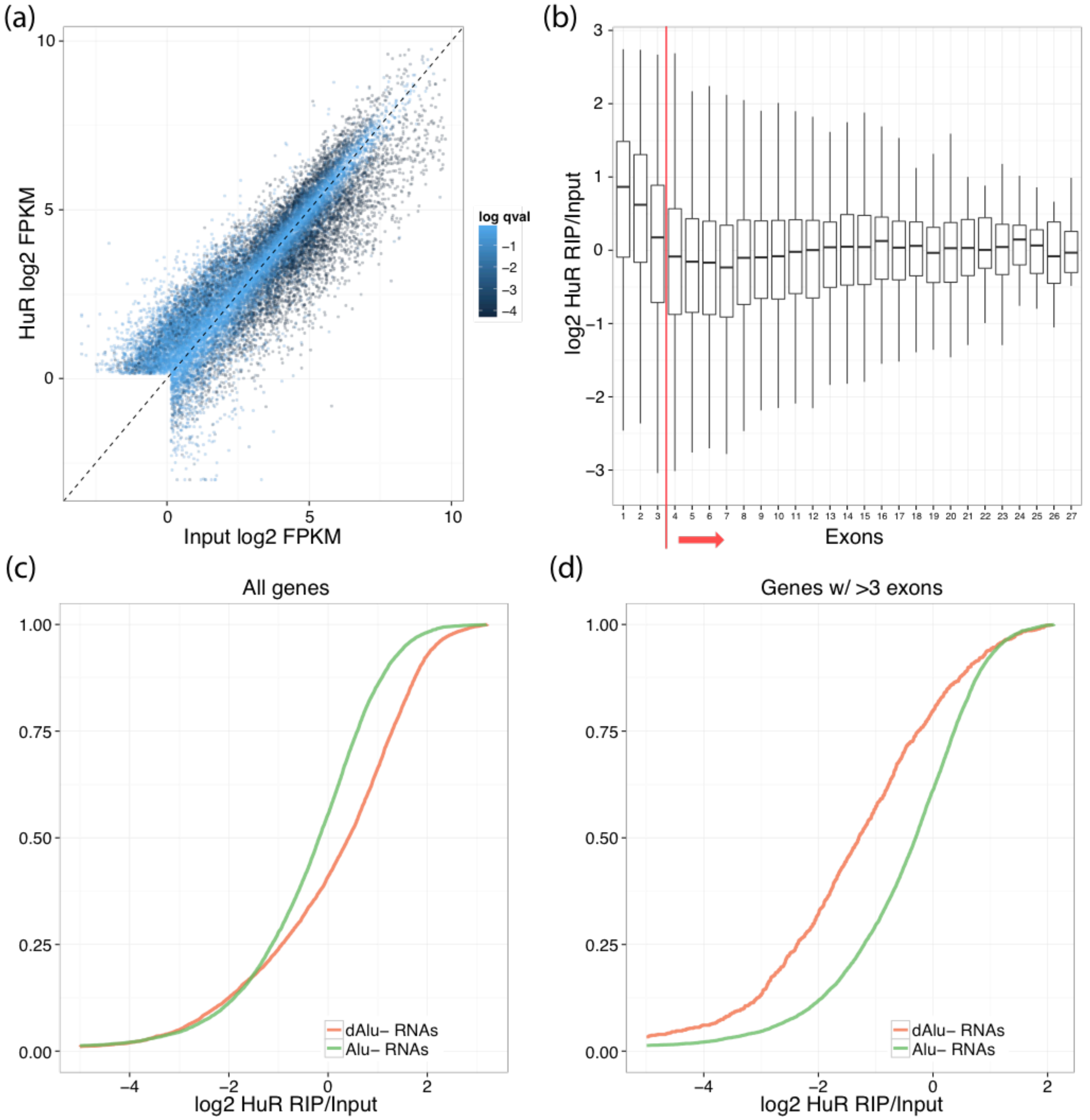

Supplementary Figure 6: Genes with antisense Alu in exons or introns are enriched in *HuR* fRIP-Seq.

(a) We performed a formaldehyde RIP-Seq for *HuR*. (b) Genes with 1-3 exons had higher fold changes than genes with >3 exons, shown as the distributions of the fold changes for all genes with each number of exons. The box plots the interquartile range (IQR) of the distribution, with the middle line marking the median. The upper/lower whiskers extend from the top/bottom of the box to the highest/lowest value that is within 1.5 * IQR of the box. (c) Thus, when classifying genes as having an antisense Alu (Alu- RNAs) or being devoid (dAlu- RNAs), the strong correlation between exon number and probability of having an Alu (because most Alu's are in introns) overwhelms our question of interest and manifests as unexpected lower fold changes for Alu- RNAs. (d) However, considering only genes with >3 exons, we again see strong evidence that Alu- RNAs Alu are bound by *HuR*.

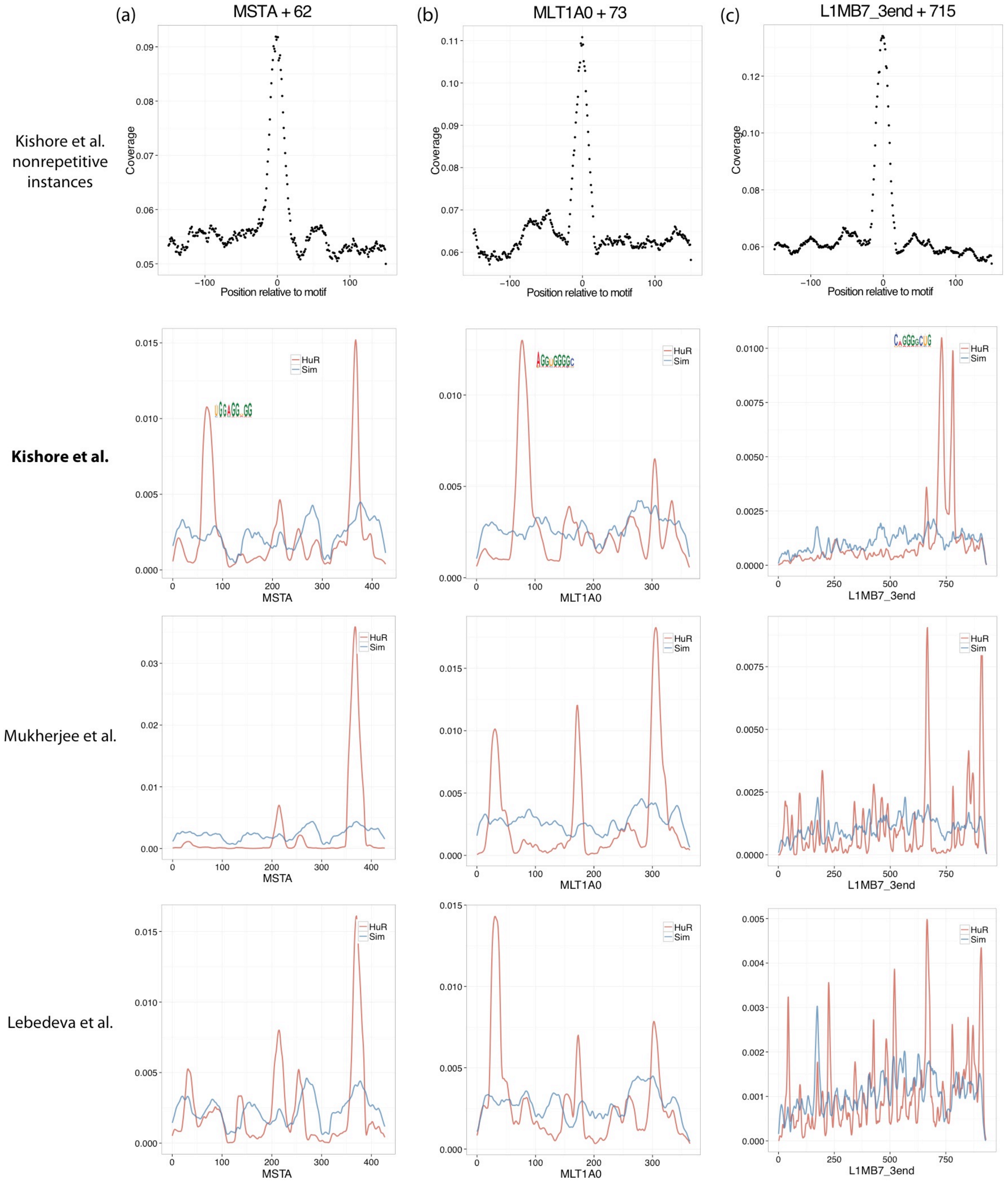

Supplementary Figure 7: Noncanonical Kishore et al. *HuR* TE-specific motifs have enrichment of CLIP-Seq alignments.

CLIP-Seq alignment coverage for noncanonical *HuR* TESMs in (a) MSTA, (b) MLT1A0, and (c) L1MB7 discovered in *HuR* CLIP-Seq by Kishore et al. (Top panel) Kishore et al. coverage on nonrepetitive instances of these motifs strongly validates the affinity of *HuR* for these motifs. (Kishore panel) Kishore et al. coverage along the TE consensus sequences shows clear peaked coverage on the TESMs. (Mukherjee and Lebedeva panels) *HuR* CLIP-Seq performed by

Mukherjee et al. and Lebedeva et al. do not share the same coverage peaks, but do have very nearby upstream peaks in MLT1A0 and L1MB7.

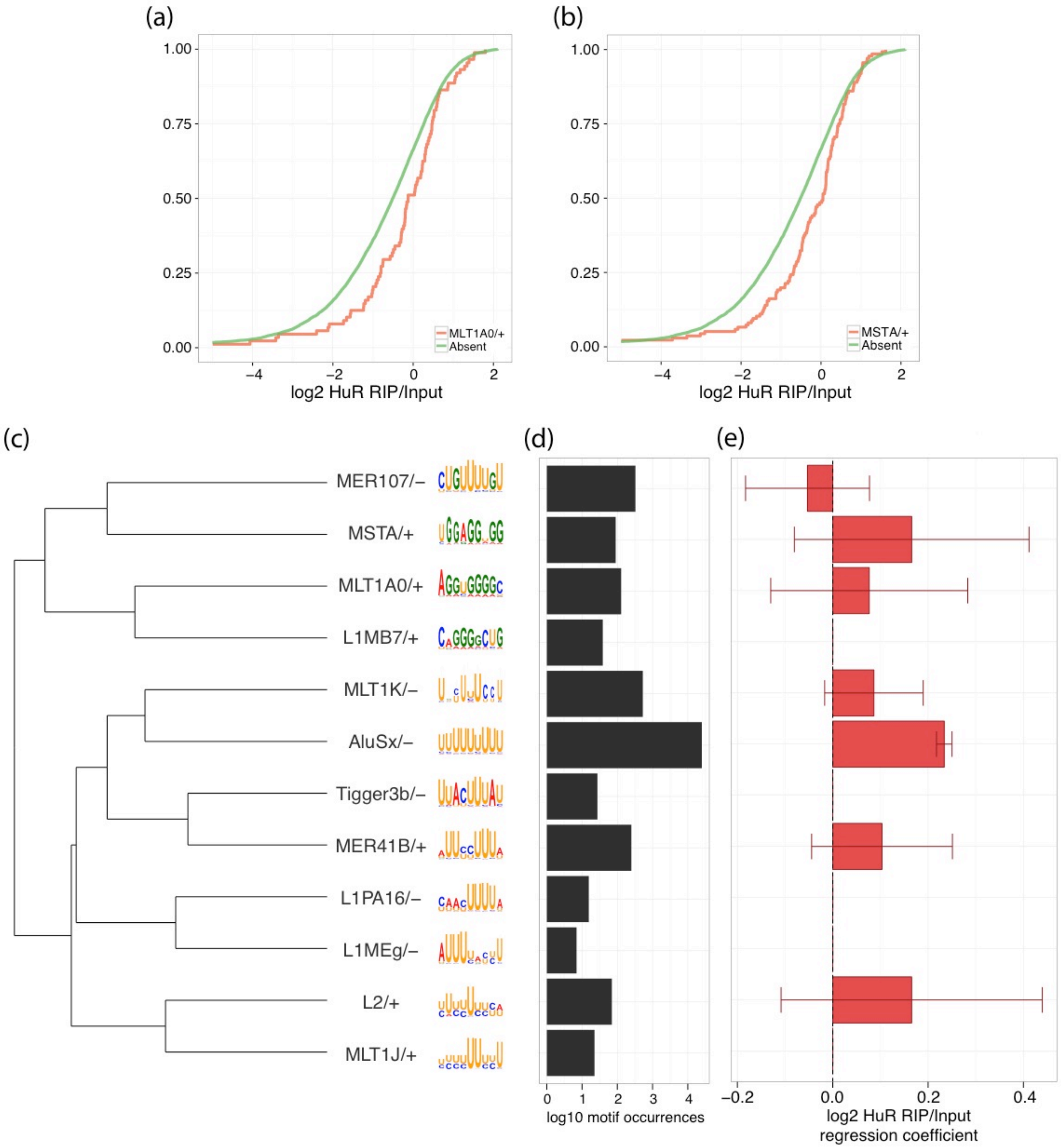

Supplementary Figure 8: *HuR* formaldehyde RIP-Seq suggests most motifs are valid. (c,d) As in Figure 3, we collapsed highly redundant motifs into twelve representatives and hierarchically clustered using *information coverage Euclidean distance* (see Methods){Stegmaier:2013ch}. Even noncanonical motifs like the G-rich motifs in (a) sense MLT1A0 and (b) sense MSTA elements showed evidence of binding in the formaldehyde RIP-Seq. (e) To consider the motifs in the context of the ubiquity of AluSx U-rich motifs (d), we represented each gene as a vector of counts of motifs in each cluster in (c) and computed a linear regression against the gene fold changes. 6 out of 7 regression coefficients based on >40 motif occurrences had a positive effect on the fold change towards *HuR* binding, including 2 of 3 noncanonical motifs. However, due to the extreme prevalence of the AluSx U-rich motif, only its coefficient achieves significance in the regression.

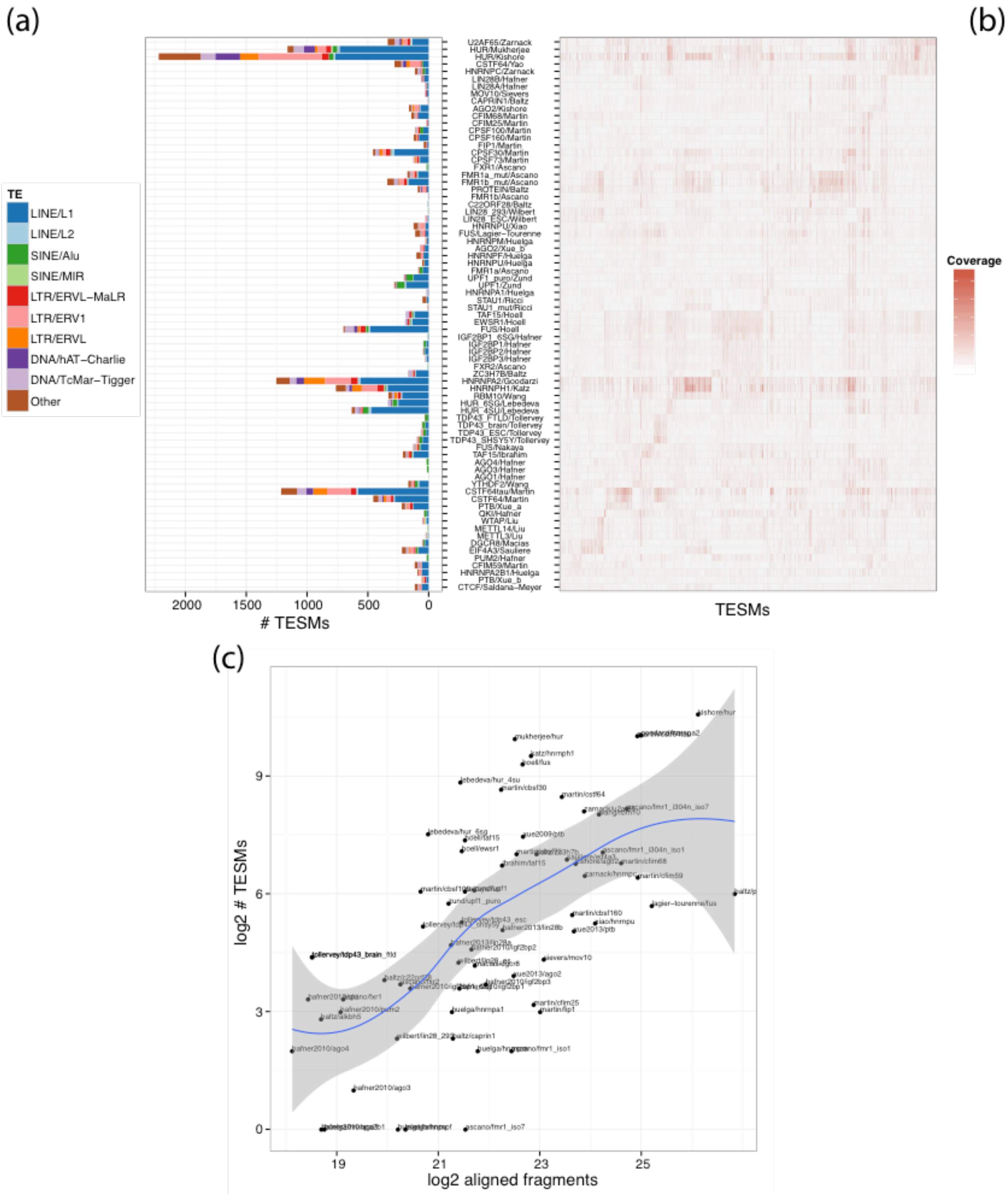

Supplementary Figure 9: Thousands of TE-specific motifs attract CLIP-Seq coverage. (a) Bars represent the number of TE-specific motifs discovered for each dataset, broken down by TE family, (c) which depends on the sequencing depth. (b) We plotted a coverage statistic for all TE-specific motifs comparing the coverage at the motif to the mean coverage in a surrounding 200 nt window. Clustering the datasets by their TESM coverage profiles revealed RBPs with similar binding preferences. The *HuR* datasets clustered together and shared their affinity for well-studied AU-rich elements{Barreau:2005dx} with many other RBPs, including *hnRNP C* and *U2AF65.* Hafner et al. detected an enriched CAUH motif for three members of the

IGF2BP family, explaining their nearby clustering{Hafner:2010kr}. FET family proteins also clustered with AU binders, matching the observations of Hoell et al., who verified binding of *FUS* to these elements using electrophoretic mobility shift assays{Hoell:2011dg}. Further establishing the validity of these RBP-TE interactions as being above the background bias of the experiment, we found that RBPs assayed in the two largest datasets collected by Hafner et al.{Hafner:2010kr} and Martin et al.{Martin:2012cn}, clustered together only when the RBPs belonged to the same family and diverged otherwise.

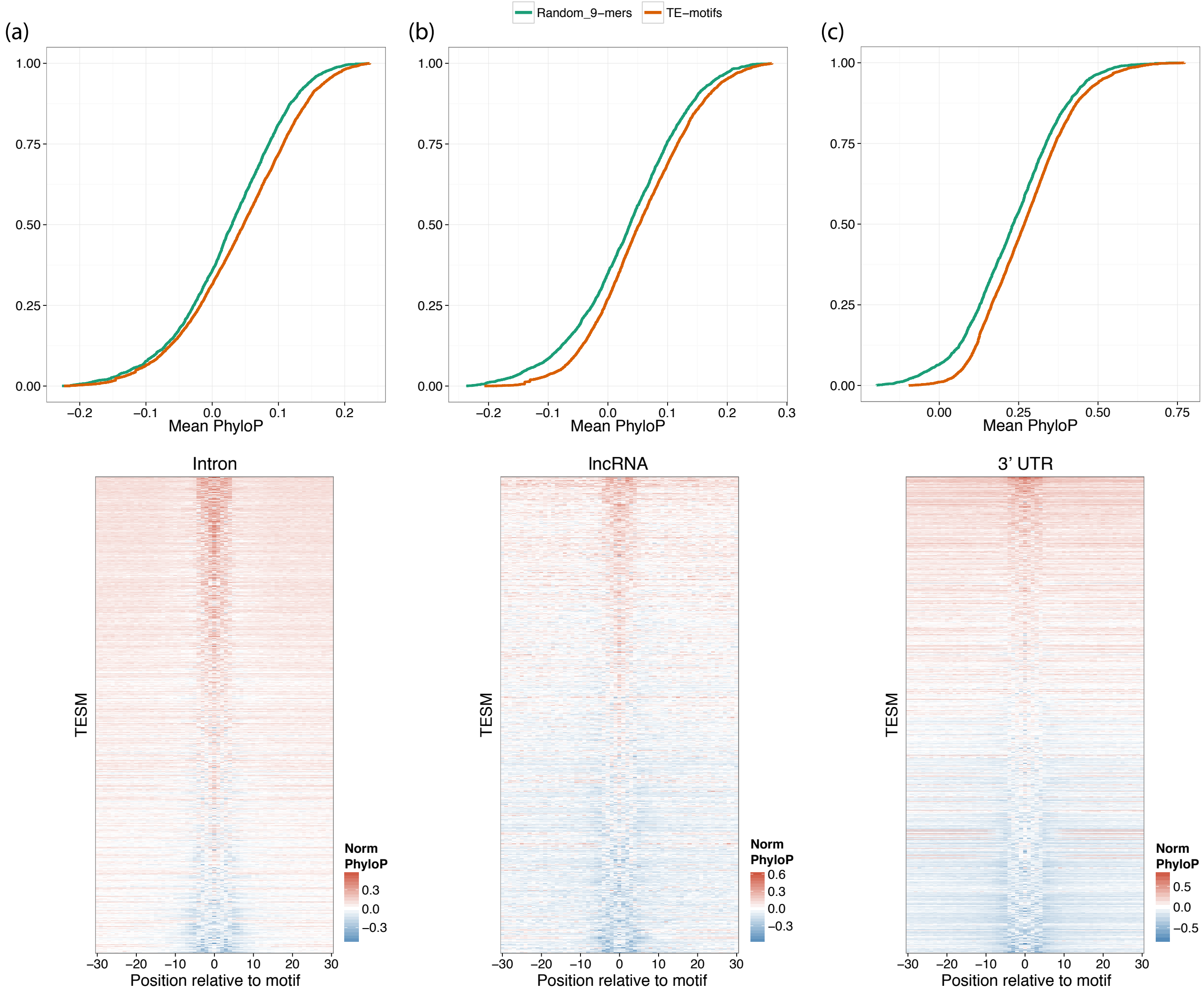

Supplementary Figure 10: TE-specific motifs are significantly conserved.
The distributions of mean PhyloP score annotating occurrences of every TE-specific motif are significantly greater than samples of random 9-mers from the transcriptome-wide 9-mer distribution in (a) introns, (b) lncRNAs, and (c) 3’ UTRs. The heatmaps in the bottom panel plot the median PhyloP score across all motif occurrences in that annotation class normalized by subtracting the background median PhyloP score for that annotation class.

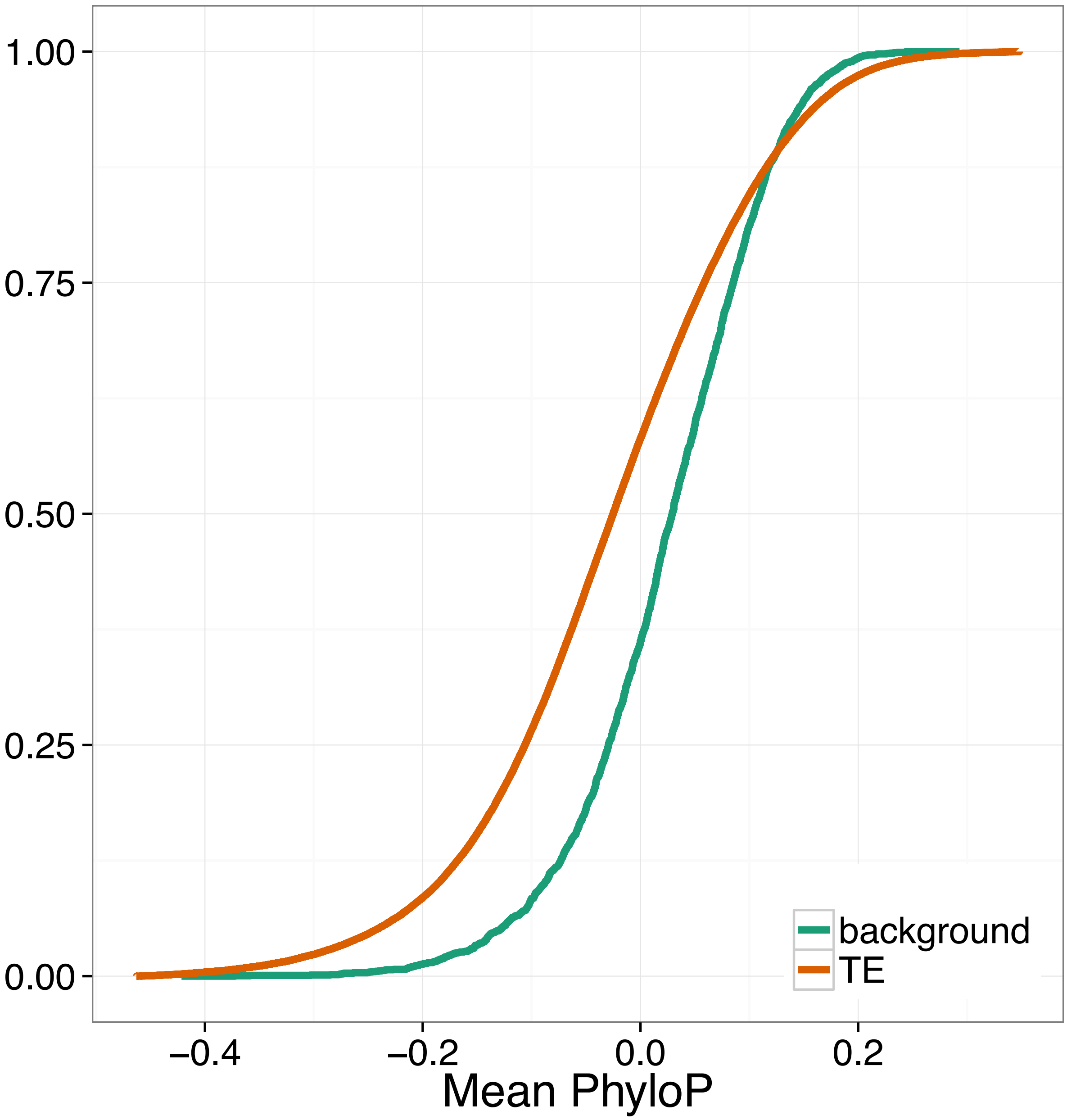

Supplementary Figure 11: TE 9-mers are less conserved in introns.
The distribution of mean intron PhyloP score for 9-mers present in consensus TE sequences is much less than the distribution for 9-mers chosen at random from the transcriptome-wide 9-mer distribution. TE consensus sequences were obtained from DFAM profiles.

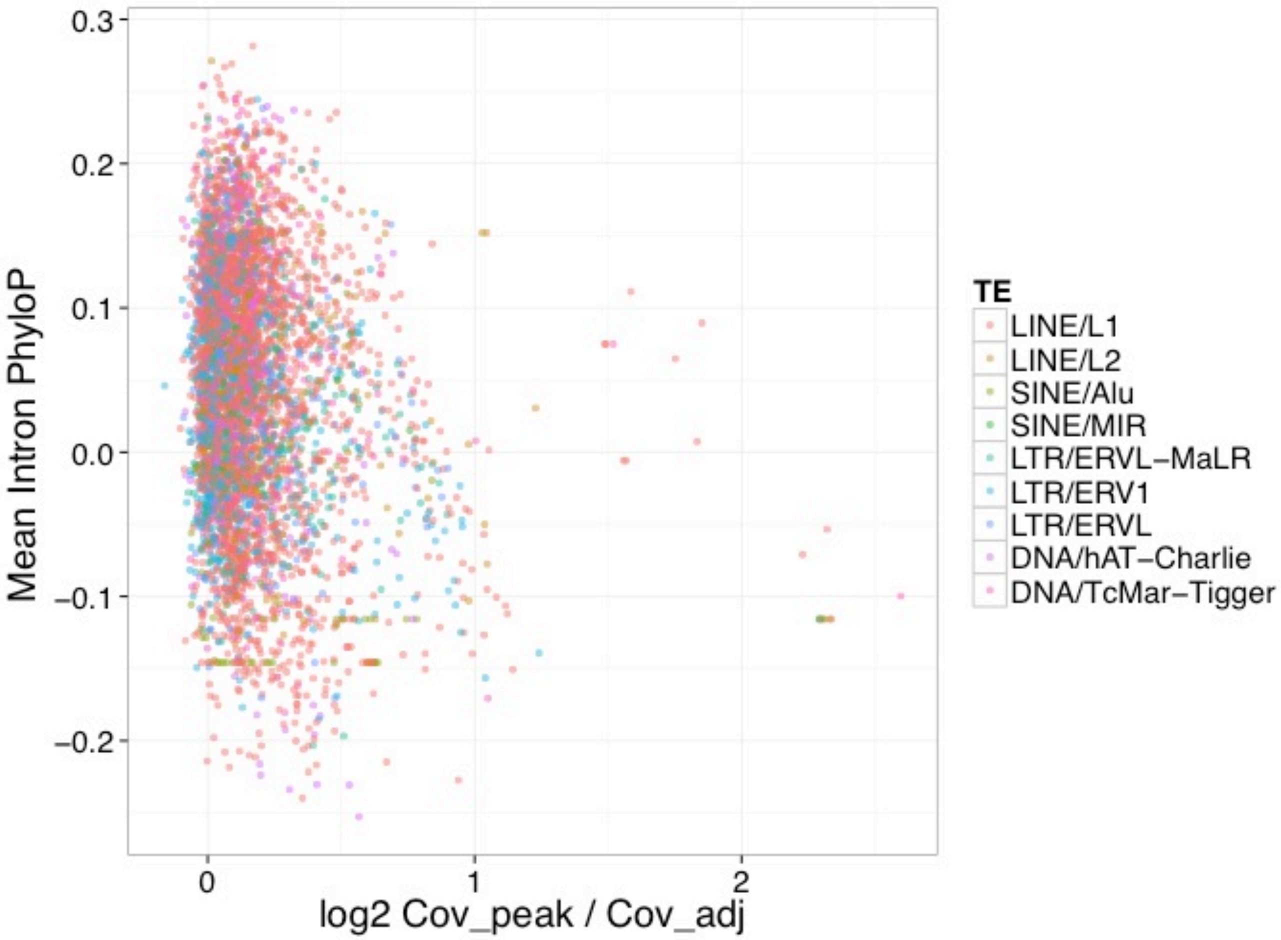

Supplementary Figure 12: TE-specific motif nonrepetitive coverage and conservation appear unrelated.
Plotting a coverage peak statistic of the CLIP-Seq alignment coverage at the motif normalized by the coverage of a surrounding 200 nt window versus the mean PhyloP score of the motif occurrences in introns demonstrates no apparent relationship between coverage and conservation.

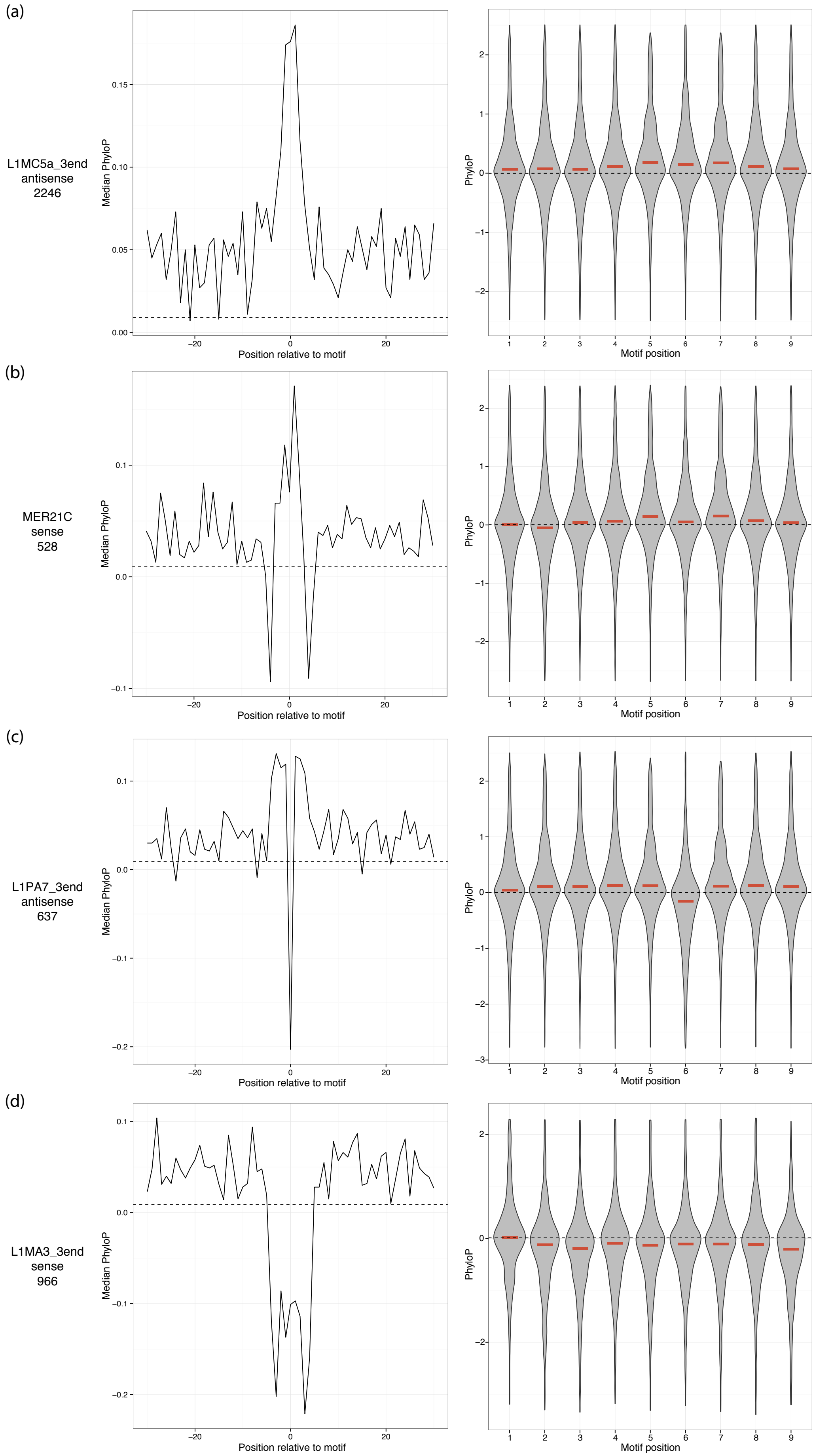

(a)
L1MC5a_3end
antisense
2246
Median PhyloP
Position relative to motif
PhyloP
Motif position
(b)
MER21C
sense
528
Median PhyloP
Position relative to motif
PhyloP
Motif position
(c)
L1PA7_3end
antisense
637
Median PhyloP
Position relative to motif
PhyloP
Motif position
(d)
L1MA3_3end
sense
966
Median PhyloP
Position relative to motif
PhyloP
Motif position

Supplementary Figure 13: Conservation is nonuniform across TE-specific motif positions.

PhyloP distributions for TE-specific motif occurrences at nucleotide resolution revealed nonuniform conservation. In the left panels, we plotted the distribution medians in a 60 nt window. In the right panels, we plotted the distributions as violin plots for the motif only. (a) Many motifs had high conservation scores across the motif. (b,c) Others had more variable patterns of positions mutating faster or slower than average. (d) A final set had a high mutation rate.

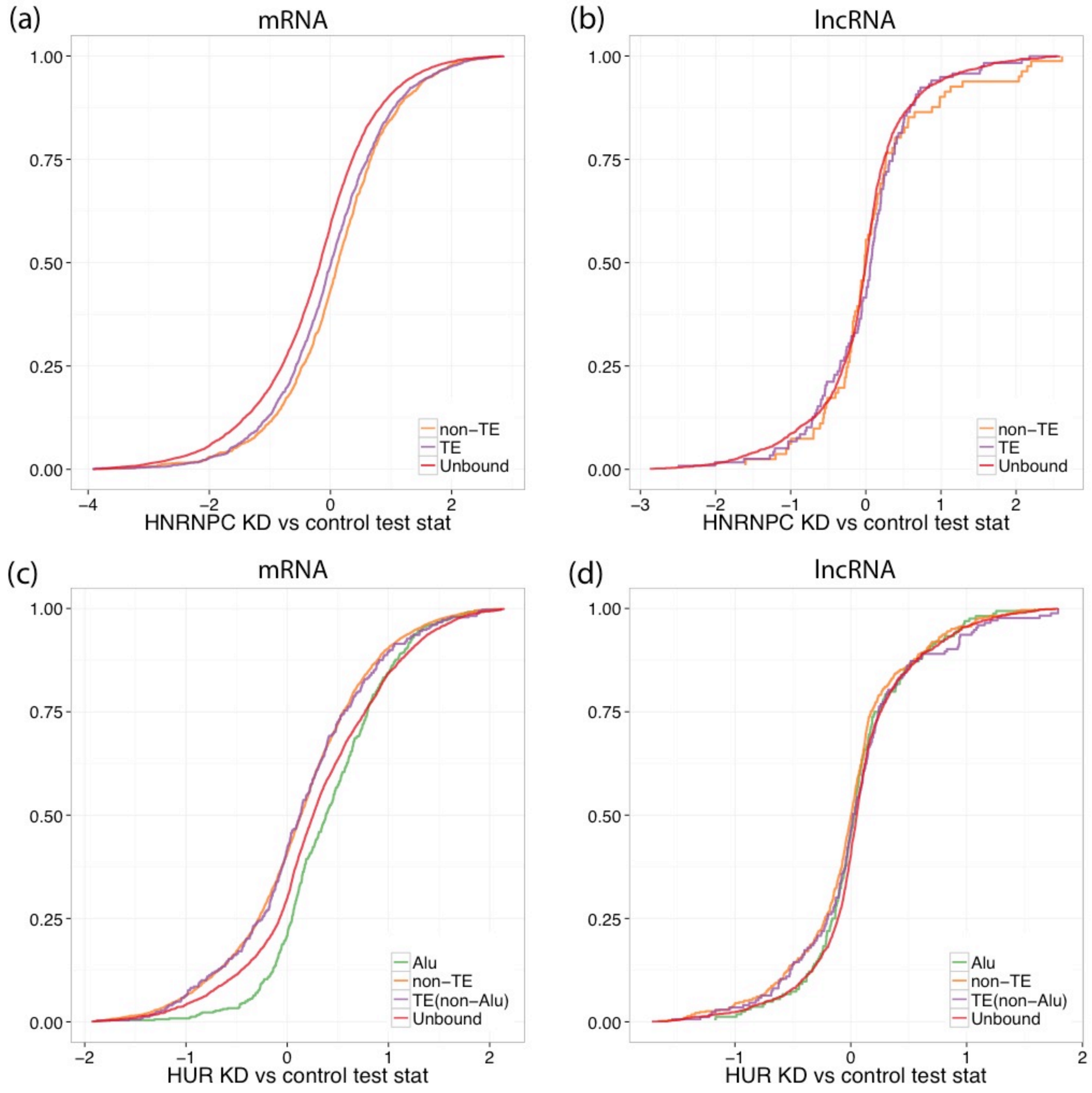

Supplementary Figure 14: TE and non-TE sites affect genes similarly in both mRNAs and lncRNAs.

(a) mRNAs and (b) lncRNAs targeted by *hnRNP* C only in TE sites are similarly upregulated to genes targeted only in nonrepetitive sites, shown here as the cumulative distributions of the Cuffdiff differential expression test statistic for genes bound only in non-TE sites, only in TE sites, and unbound. The same is true for (c) mRNAs and (d) lncRNAs targeted by *HuR*, as is the differential regulation via Alu sites.

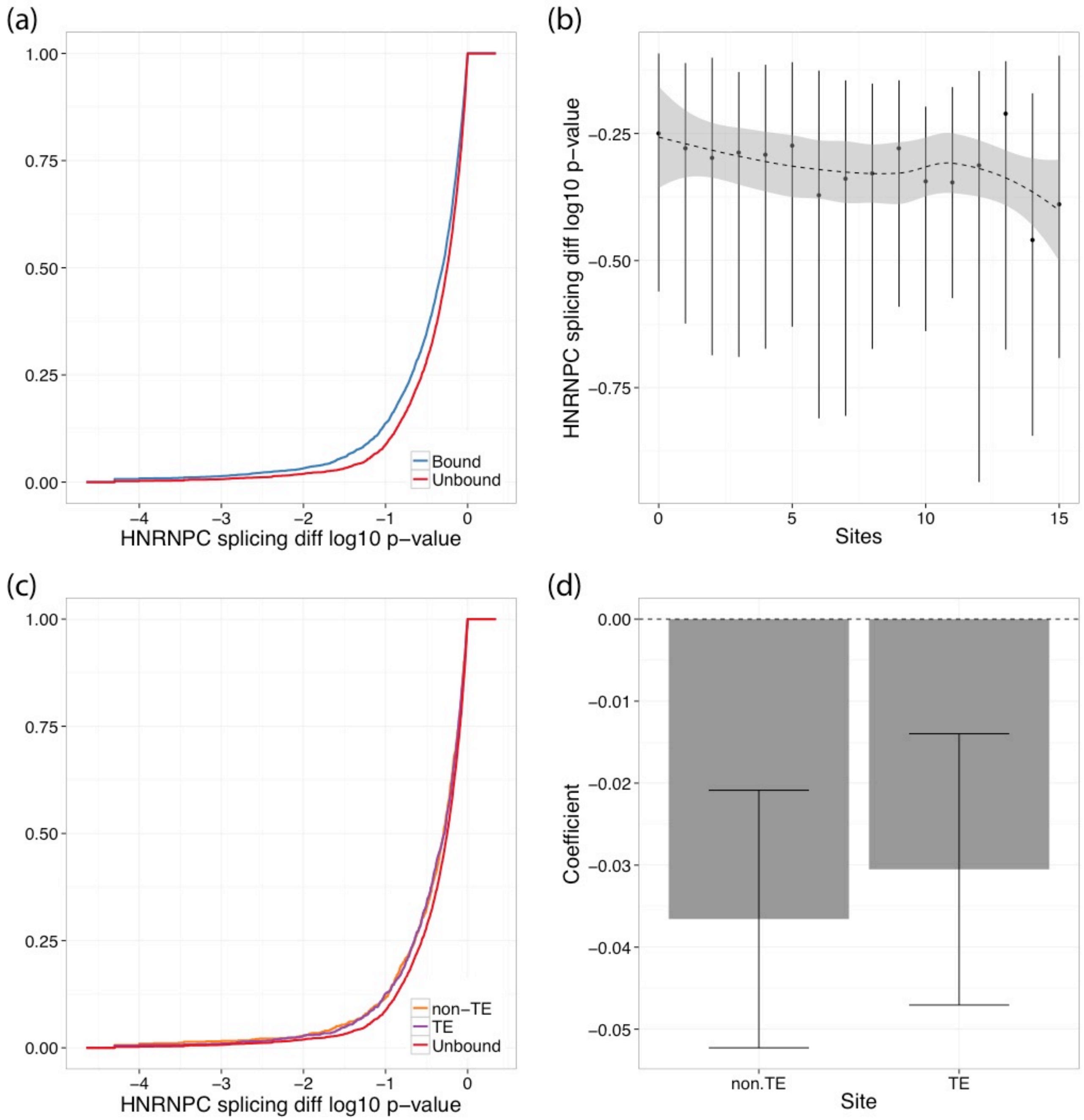

Supplementary Figure 15: *hnRNP* C-TE binding sites affect splicing similarly to nonrepetitive sites.

(a) Genes targeted by *hnRNP C* have more evidence for splicing changes, measured as the Cuffdiff p-value from a statistical test for an isoform switch. (b) More bound sites leads to more evidence for a splicing change, plotted as the medians and interquartile ranges of the distribution of splicing difference p-values. (c) Splitting the p-value distributions by genes bound only in nonrepetitive sites and genes bound only in TE sites shows that TE sites contribute similarly to this effect. (d) A linear regression on the logarithm of the number of sites in TEs and nonrepetitive sequence reiterates that both categories similarly affect splicing changes.

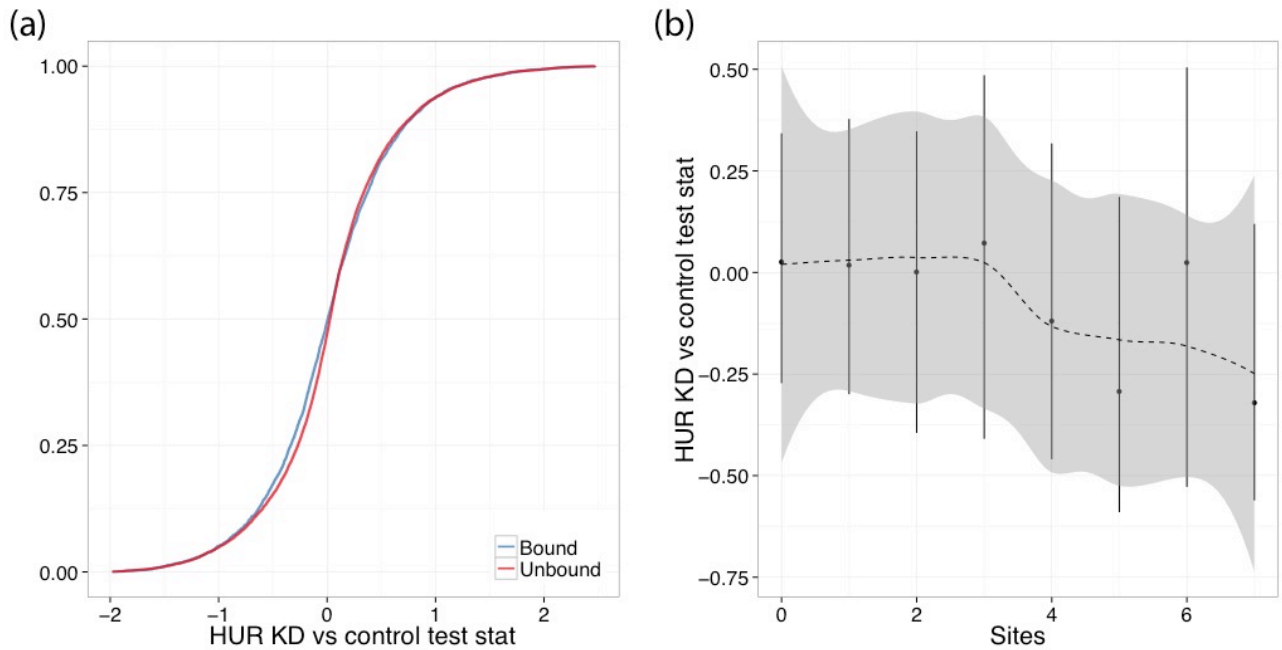

Supplementary Figure 16: Lebedeva *HuR* knockdown RNA-Seq shows statistically significant, but weak, downregulation of *HuR* bound genes.

Genes bound by *HuR* via CLIP-Seq were downregulated upon *HuR* knockdown by Lebedeva et al, shown here as the (a) cumulative distributions of the Cuffdiff differential expression test statistics for bound and unbound genes and (b) the number of binding sites plotted against the medians and interquartile ranges of the differential expression test statistics.

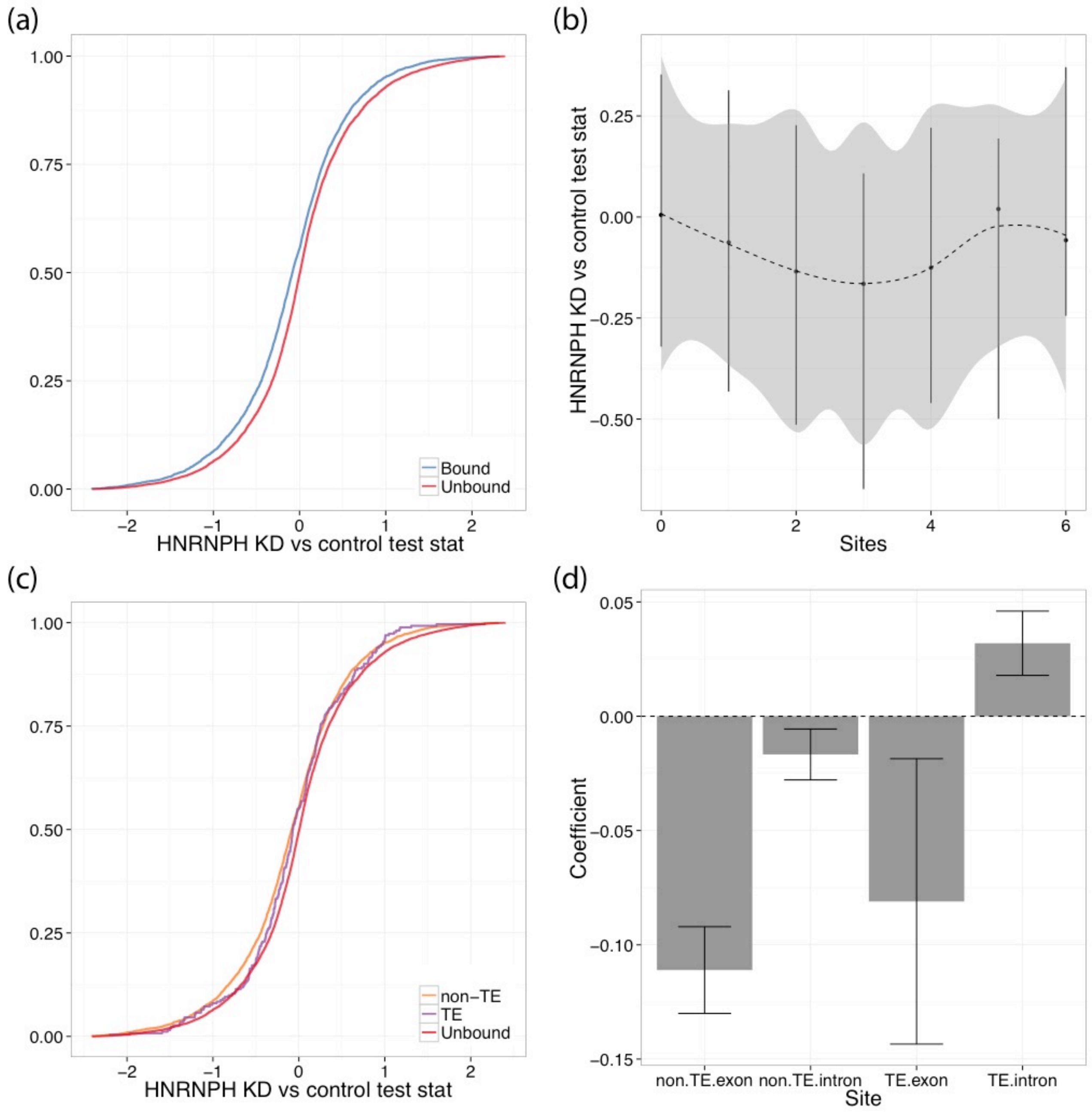

Supplementary Figure 17: *hnRNP H1* exonic TE binding sites stabilize genes similarly to nonrepetitive exonic sites.

Genes targeted by *hnRNP H1* via CLIP-Seq in exon sites were downregulated upon *hnRNP H1* knockdown, shown here as the (a) cumulative distributions of the Cuffdiff differential expression test statistics for bound and unbound genes and (b) the number of binding sites plotted against the medians and interquartile ranges of the differential expression test statistics. (c) Genes targeted only in TE sites were similarly affected as genes targeted only in non-TE sites. (d) A linear regression on the logarithm of the number of sites in each class verified that TE and nonrepetitive exon sites predicted downregulation.

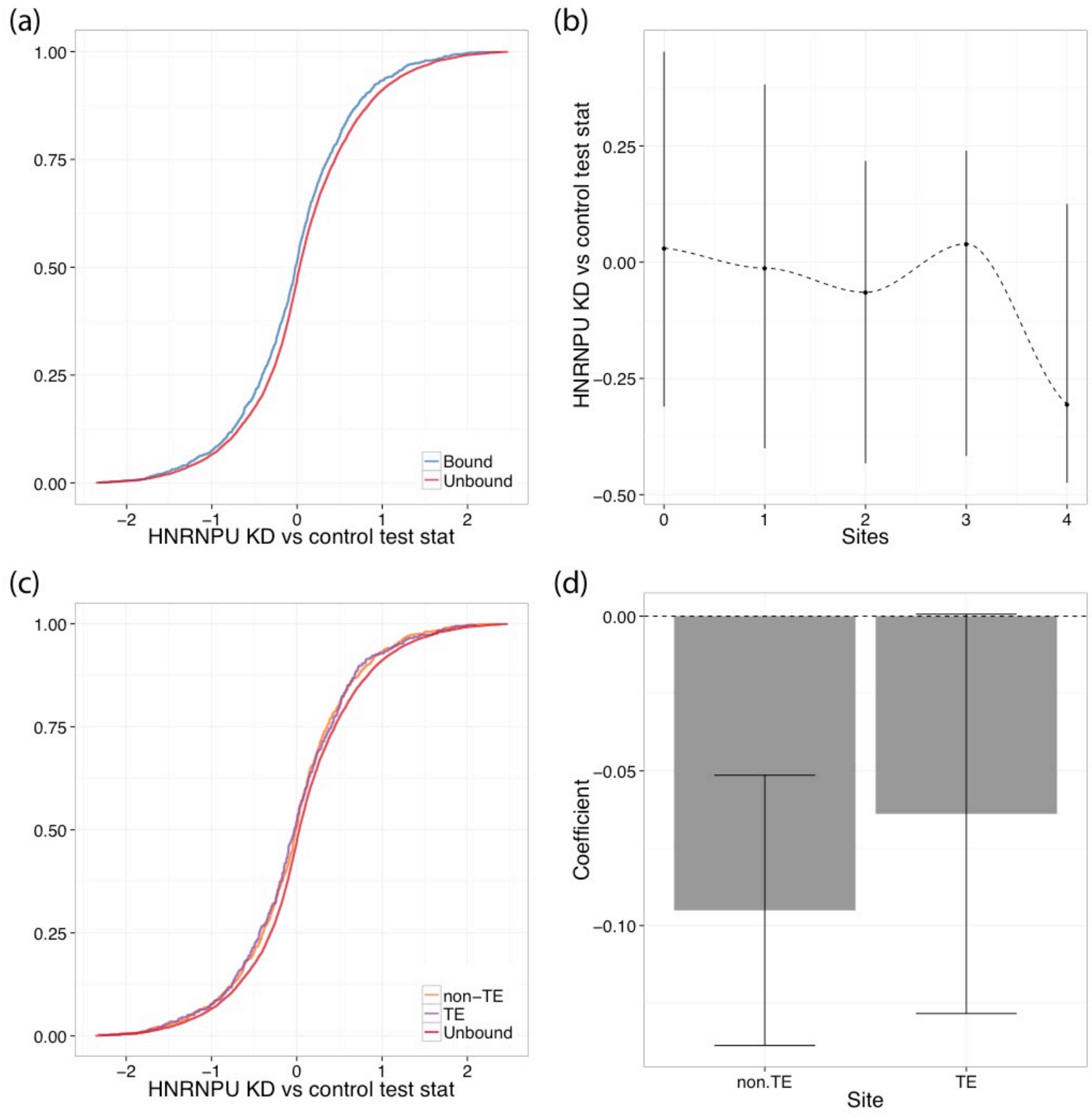

Supplementary Figure 18: *hnRNP U* TE binding sites stabilize genes similarly to nonrepetitive sites.

Genes targeted by *hnRNP U* via CLIP-Seq in exon sites were downregulated upon *hnRNP U* knockdown, shown here as the (a) cumulative distributions of the Cuffdiff differential expression test statistics for bound and unbound genes and (b) the number of binding sites plotted against the medians and interquartile ranges of the differential expression test statistics. (c) Genes targeted only in TE sites were similarly affected as genes targeted only in non-TE sites. (d) A linear regression on the logarithm of the number of sites in each class verified that both TE and nonrepetitive sites predicted downregulation.

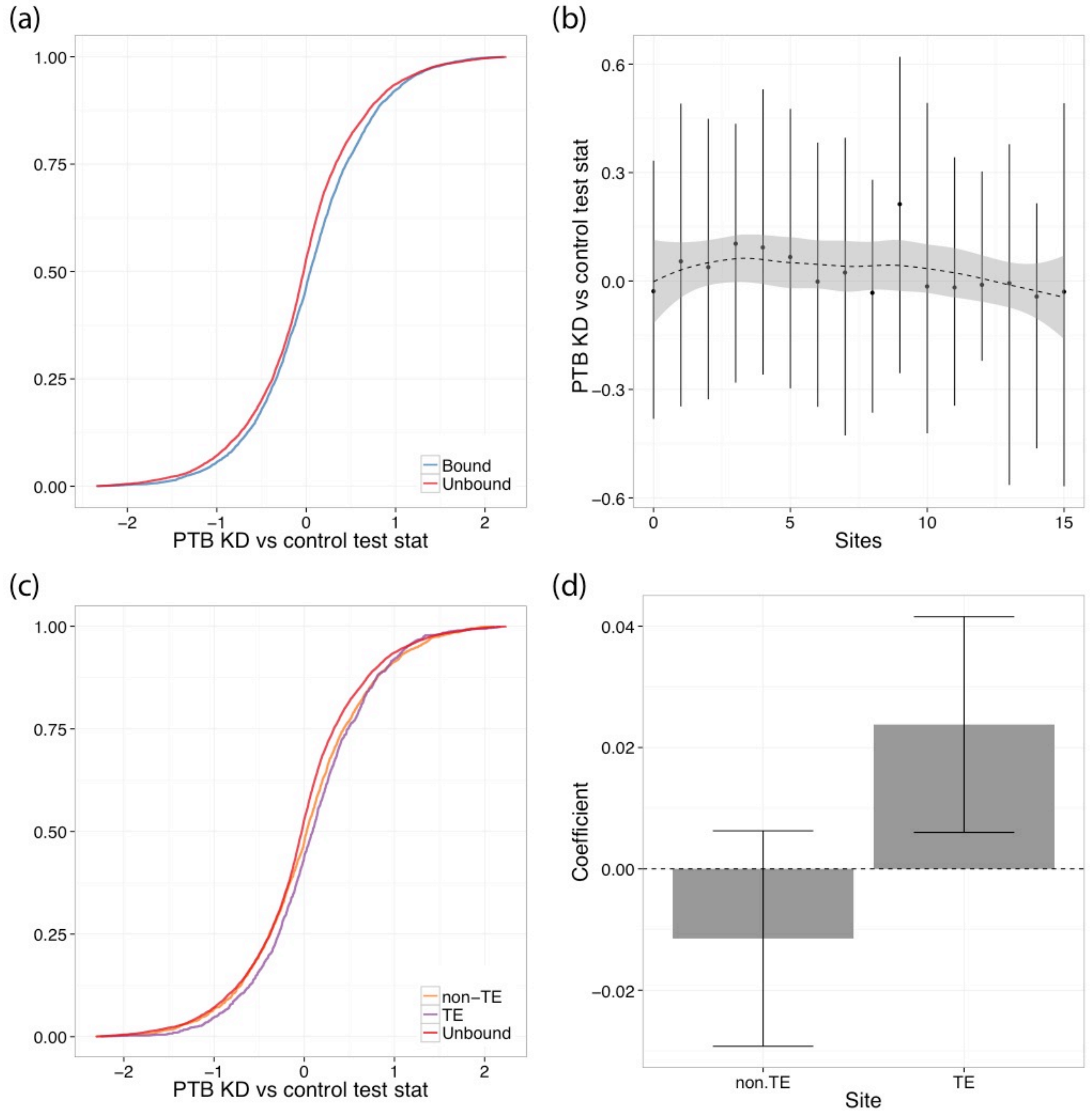

Supplementary Figure 19: *PTB* TE binding sites repress genes similarly to nonrepetitive sites.

Genes targeted by *PTB* via CLIP-Seq in exon sites were upregulated upon *PTB* knockdown, shown here as the (a) cumulative distributions of the Cuffdiff differential expression test statistics for bound and unbound genes and (b) the number of binding sites plotted against the medians and interquartile ranges of the differential expression test statistics. (c) Genes targeted only in TE sites were similarly affected as genes targeted only in non-TE sites. (d) A linear regression on the logarithm of the number of sites in each class indicates that TE sites drive the upregulation.

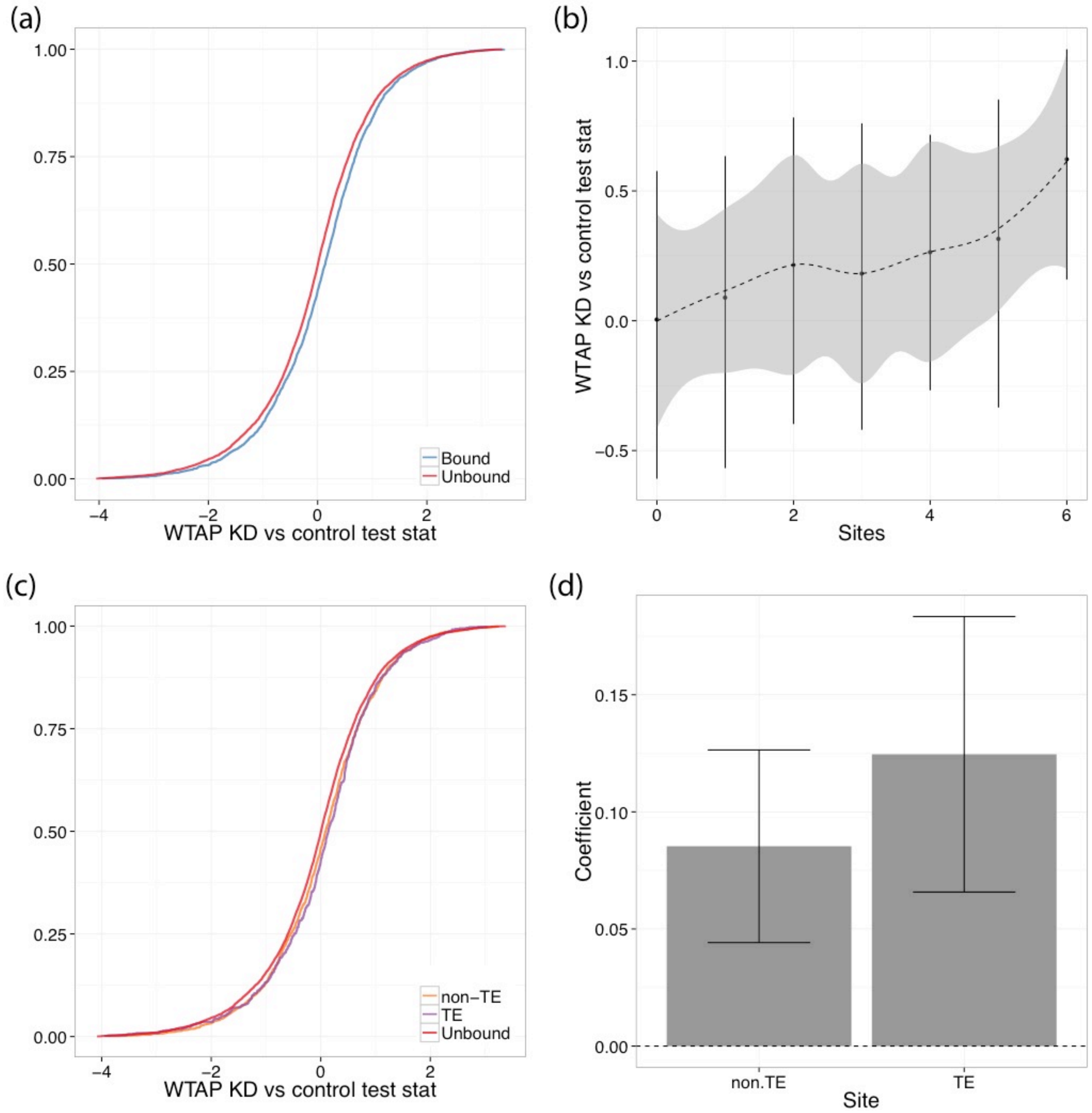

Supplementary Figure 20: *WTAP* TE binding sites repress genes similarly to nonrepetitive sites.
Genes targeted by *WTAP* via CLIP-Seq in exon sites were upregulated upon *WTAP* knockdown, shown here as the (a) cumulative distributions of the Cuffdiff differential expression test statistics for bound and unbound genes and (b) the number of binding sites plotted against the medians and interquartile ranges of the differential expression test statistics. (c) Genes targeted only in TE sites were similarly affected as genes targeted only in non-TE sites. (d) A linear regression on the logarithm of the number of sites in each class verified that both TE and nonrepetitive sites predicted upregulation.

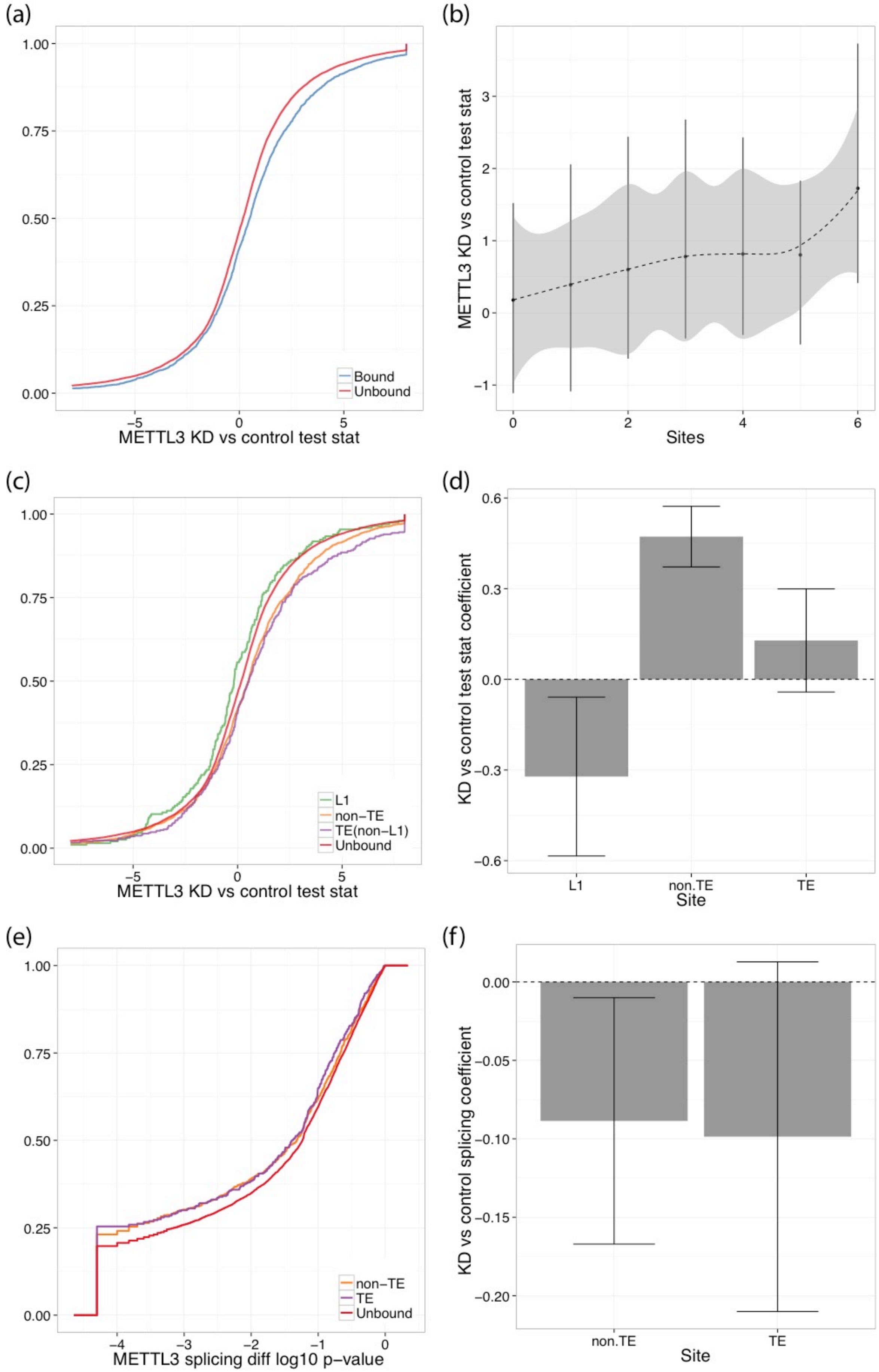
(a)
1.00
0.75
0.50
0.25
0.00
−5
0
5
Bound
Unbound
METTL3 KD vs control test stat
(b)
METTL3 KD vs control test stat
3
2
1
0
−1
0
2
4
6
Sites
(c)
1.00
0.75
0.50
0.25
0.00
−5
0
5
L1
non−TE
TE(non−L1)
Unbound
METTL3 KD vs control test stat
(d)
KD vs control test stat coefficient
0.6
0.3
0.0
−0.3
−0.6
L1
non.TE
TE
Site
(e)
1.00
0.75
0.50
0.25
0.00
−4
−3
−2
−1
0
non−TE
TE
Unbound
METTL3 splicing diff log10 p−value
(f)
KD vs control splicing coefficient
0.00
−0.05
−0.10
−0.15
−0.20
non.TE
TE
Site

Supplementary Figure 21: *METTL3* TE binding sites repress genes similarly to nonrepetitive sites, unless in L1 elements.
Genes targeted by *METTL3* via CLIP-Seq in exon sites were upregulated upon *METTL3* knockdown, shown here as the (a) cumulative distributions of the Cuffdiff differential expression test statistics for bound and unbound genes and (b) the number of binding sites plotted against the medians and interquartile ranges of the differential expression test statistics. (c) Genes targeted only in non-L1 TE sites were similarly affected as genes targeted only in non-TE sites, but genes targeted in only L1 sites were downregulated upon knockdown. (d) A linear regression on the logarithm of the number of sites in each class verified that both non-L1 TE and nonrepetitive sites predicted upregulation, but L1 sites did not. (e) *METTL3* binding also affects splicing changes, similarly in TE and nonrepetitive sites, shown here as the distributions of Cuffdiff p-value from a statistical test for an isoform switch. (f) A linear regression on the logarithm of the number of sites in TEs and nonrepetitive sequence reiterates that both categories similarly affect splicing changes.
